# Genome sequencing reveals fine scale diversification and reticulation history during speciation in *Sus*

**DOI:** 10.1186/gb-2013-14-9-r107

**Published:** 2013-09-26

**Authors:** Laurent AF Frantz, Joshua G Schraiber, Ole Madsen, Hendrik-Jan Megens, Mirte Bosse, Yogesh Paudel, Gono Semiadi, Erik Meijaard, Ning Li, Richard PMA Crooijmans, Alan L Archibald, Montgomery Slatkin, Lawrence B Schook, Greger Larson, Martien AM Groenen

**Affiliations:** 1 Animal Breeding and Genomics Group, Wageningen University, De Elst 1, Wageningen, WD 6708, The Netherlands; 2 Department of Integrative Biology, University of California, Berkeley, CA 94720-3140, USA; 3 Puslit Biologi LIPI, Jl. Raya Jakarta-Bogor Km. 46, Cibinong 16911, Jawa Barat, Indonesia; 4 People and Nature Consulting International, Vila Lumbung House no. 6, Jl. Kerobokan Raya 1000x, Badung 80361, Bali, Indonesia; 5 School of Archaeology and Anthropology, Australian National University, Canberra ACT 0200, Australia; 6 State Key Laboratory for Agrobiotechnology, China Agricultural University, Beijing 100193, PR China; 7 The Roslin Institute and Royal (Dick) School of Veterinary Studies, University of Edinburgh, Easter Bush, Midlothian EH25 9RG, UK; 8 Department of Animal Sciences, University of Illinois, Urbana-Champaign, Illinois 61801, USA; 9 Durham Evolution and Ancient DNA, Department of Archaeology, Durham University, Durham DH1 3LE, UK

## Abstract

**Background:**

Elucidating the process of speciation requires an in-depth understanding of the evolutionary history of the species in question. Studies that rely upon a limited number of genetic loci do not always reveal actual evolutionary history, and often confuse inferences related to phylogeny and speciation. Whole-genome data, however, can overcome this issue by providing a nearly unbiased window into the patterns and processes of speciation. In order to reveal the complexity of the speciation process, we sequenced and analyzed the genomes of 10 wild pigs, representing morphologically or geographically well-defined species and subspecies of the genus *Sus *from insular and mainland Southeast Asia, and one African common warthog.

**Results:**

Our data highlight the importance of past cyclical climatic fluctuations in facilitating the dispersal and isolation of populations, thus leading to the diversification of suids in one of the most species-rich regions of the world. Moreover, admixture analyses revealed extensive, intra- and inter-specific gene-flow that explains previous conflicting results obtained from a limited number of loci. We show that these multiple episodes of gene-flow resulted from both natural and human-mediated dispersal.

**Conclusions:**

Our results demonstrate the importance of past climatic fluctuations and human mediated translocations in driving and complicating the process of speciation in island Southeast Asia. This case study demonstrates that genomics is a powerful tool to decipher the evolutionary history of a genus, and reveals the complexity of the process of speciation.

## Background

The diversity of life on Earth owes its existence to the process of speciation. The emergence of genetic techniques has allowed the relationships amongst hundreds of species to be investigated, and DNA studies have been invaluable in resolving long-standing taxonomic and phylogenetic questions (for example, [1,2]. The use of limited numbers of genomic markers, however, can result in misleading impressions of the phylogenetic relationships between organisms [3]. In addition, traditional bifurcating trees are constructed on the presumption that little or no gene-flow occurs following a split between two species, though gene-flow has been shown to occur during the splits between species [4,5]. The recent advent of high-throughput sequencing allows inferences to be drawn from near-complete genomes, in turn offering an unprecedented understanding of organismal evolutionary history. The commensurate increase in resolving power has allowed numerous questions to be addressed, including those related to genomic structure, deep phylogenetic relationships, the genetic variation responsible for specific phenotypes, and hybridization patterns between ancient hominids [6,7]. Few studies, however, have taken advantage of complete genomes to investigate the process of speciation.

**Figure 1:**
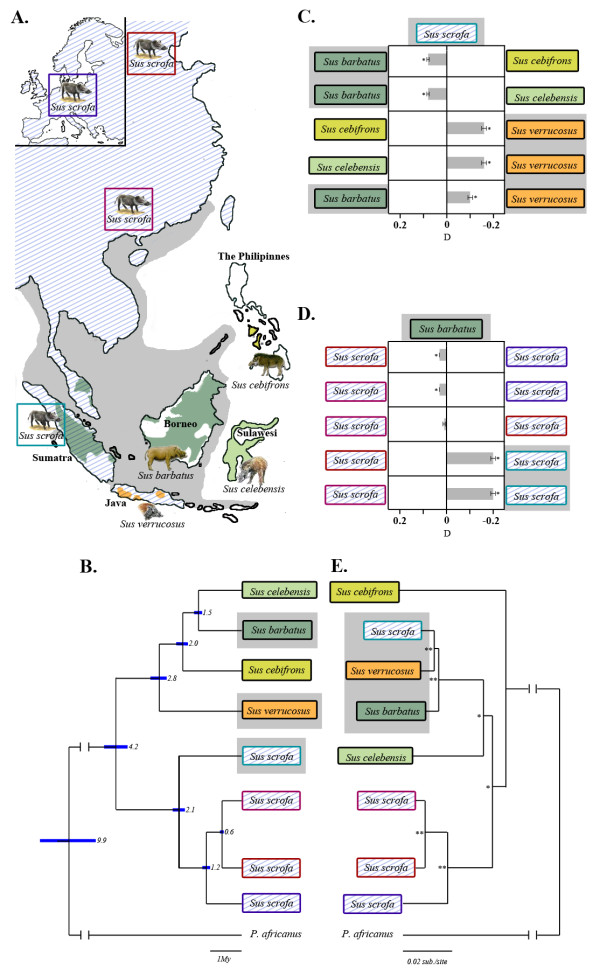
**Geographic distribution, phylogenetic relationships and admixture between *Sus *lineages**. **(A) **A map of Island and Mainland Southeast Asia depicting the modern distributions of five *Sus *species. The grey shaded area represents the maximum geographical extent of Sundaland during periods of low sea level. **(B) **Phylogenetic relationships among *Sus *species inferred from nuclear DNA. Node labels show age in millions of years and 95% confidence interval. Grey shading highlights taxa living on Sundaland **(C,D) **Diagrams depicting the excess derived allele sharing when comparing sister taxa and outgroups. Each row contains the fraction of excess allele sharing by a taxon (left/right) with the top label/outgroup (*S. scrofa *or *S. barbatus*) relative to its sister taxon (left/right). The grey bar points in the direction of the taxon that shares more derived alleles with the outgroup than its sister taxon, and its magnitude indicates the amount of excess (D). Black bars represent 1 standard error and stars indicate D values significantly different from 0 (*P *< 0.01; see Materials and methods). **(E) **A mitochondrial DNA Bayesian phylogenetic-based tree with node labels that represent posterior probabilities (* > 0.85; ** = 1).

Wallace [8] first recognized that Island Southeast Asia (ISEA) is an ideal natural laboratory to study speciation. Over the past 50 million years (My) tectonic activity has considerably altered the geography of this region. In addition, large-scale climatic fluctuations beginning in the early Pliocene [9] affected the region's biogeography [10]. Successive glacial and interglacial periods lowered and raised sea levels, thus alternately separating and connecting large landmasses. During cold periods, the Malay Peninsula, Borneo, Sumatra and Java formed the contiguous landmass known as Sundaland (Figure 1A), while in warmer periods these islands were isolated from each other. These alternating climatic conditions required frequent adaptation and induced intermittent allopatric and parapatric speciation processes. The fluctuations also created an ideal environment for diversification that has resulted in a complex and species-rich assemblage [10]. The development of models that explain the process of speciation in ISEA has been further complicated by anthropogenic factors that have influenced the dispersal and distribution of numerous species in the region [11].

The five biodiversity hotspots found in ISEA and Mainland Southeast Asia (MSEA) [12] are host to at least seven morphologically defined species of pig in the genus *Sus *[13]. Aside from *Sus scrofa *(Eurasian wild boar and domestic pigs), which is distributed across most of Eurasia and parts of northern Africa, all other species of the genus *Sus *are restricted to MSEA and ISEA (Figure 1A). Because these species are still capable of interbreeding and producing fertile offspring [14], the genus *Sus *presents an excellent model to study on-going speciation. Moreover, previous studies have found discrepancies between and among the phylogenies inferred from morphological and mitochondrial DNA (mtDNA) markers [13,15,16]. Thus, the phylogeny of these species remains controversial. These discrepancies could be explained by either gene-flow between sympatric populations of different species or a rapid radiation that would have left little power to resolve the phylogeny.

The lack of a post-zygotic reproductive barrier in pigs is not an isolated case. Indeed, many vertebrate taxa, recognized as different species, can still interbreed and produce fertile offspring. For example, it has been claimed that approximately 6% of European mammalian species can interbreed with at least one other species [17]. Additionally, while most of these species are young, there are examples of interbreeding species of birds that diverged over 55 million years ago (Mya) [18]. Given the ease with which numerous closely related (and some distantly related) species can interbreed, it is important to develop and test methods that are not only robust to inter-specific gene-flow, but can also identify it. Speciation with gene-flow is expected to result in a richer phylogenetic history including periods of divergence (bifurcations) and periods of secondary contact (reticulations), and thus should leave genomic signatures.

In order to investigate the speciation history of these suids, and to assess the usefulness of whole-genome sequences to infer complex evolutionary histories, we sequenced and analyzed the complete genomes of 11 individual pigs representing five *Sus *species and an African common warthog (*Phacochoerus africanus*; Table S1 in Additional file 1). Our analysis of these 11 genomes demonstrates the power afforded by genomics to resolve a complex and controversial evolutionary history involving multiple reticulation events.

## Results

### SNP discovery and general divergence pattern across the genomes

We aligned between 153 and 566 million reads per sample to the *S*. *scrofa *reference genome (Sscrofa10.2) [19], resulting in an average read depth of 7.5 to 24× (Table S2 in Additional file 1; Materials and methods). The number of SNPs discovered in each genome sequence (Table S2 in Additional file 1) was higher in the *Sus *species than between *S. scrofa *individuals, most of which were fixed differences between the *S. scrofa *reference genome and the other species analyzed. In order to understand how substitution rate within the genus varies across the genome, we computed the average sequence divergence from the Warthog to each *Sus *species in 1 Mb windows (Materials and methods). Our results demonstrated that the average sequence divergence to the outgroup (warthog) was positively correlated with recombination rate (as estimated in *S. scrofa *[20]; tau = 0.40, *P *< 0.001), suggesting a relationship between recombination and divergence rate, as observed in other mammals [21,22].

### Phylogenomic analysis

Using near complete genome sequences, we applied several phylogenomic methods based on maximum likelihood (ML) implemented in RAxML 7.2 [23]. We used both supertree and supermatrix techniques (see Materials and methods for details). Briefly, the supertree methodology involves computing a single tree per genomic locus in combination with an *ad hoc *reconstruction of a consensus phylogeny from the single trees whereby the stochastic behavior of lineage sorting can be taken into account. In the supermatrix framework, a single tree is inferred from multiple loci assembled in multiple partitions.

We first identified regions in the genome, spanning a minimum of 5 kbp, that possessed less than 10% missing data (due to filtering) in all our samples (see Materials and methods for details; Table S3 in Additional file 1). We then built phylogenetic trees for every genomic bin identified and obtained a species tree using the supertree method STAR [24]. We also used a concatenation method by building multiple supermatrices. One hundred supermatrices, each spanning 1 Mbp, were assembled by randomly joining genomic bins. We then computed a phylogenetic tree using RAxML, with 100 fast bootstrap replicates, for each supermatrix.

We found that the species tree topology depicted in Figure 1B was the most common across all of the genomic bins analyzed (Additional file 2), but several alternative topologies appeared in substantial numbers (Additional file 3). This result is to be expected and can be caused by incomplete lineage sorting (in which deep coalescences occur in ancestral populations) and gene-flow (in which some genealogies cross species boundaries). The presence of such incongruence is created when recombination creates local gene trees; hence, we looked for a correlation between recombination rate and the frequency of alternative topologies. We found a positive correlation between mean pairwise Robinson-Foulds distance and recombination rate in 1 Mbp windows (tau = 0.53, *P *< 0.001; Materials and methods). We also found a positive correlation with mean divergence to the outgroup (tau = 0.40, *P *< 0.001). Together, these results suggest the importance of recombination in shaping the genomic landscape of speciation in suids.

To compare our results to earlier studies using mitochondrial DNA (matrilineal lineage), we carried out a Bayesian phylogenetic analysis using near-complete mitochondrial genomes (Materials and methods). The resulting topology is consistent with previous studies [15,16,25] and shows a clear discordance with the phylogenetic tree obtained from autosomal chromosomes (Figure 1B,E). This discordance is expected given the wide range of topologies found in the autosomes, especially because mitochondrial DNA represents only one locus with no recombination.

The phylogenetic discordance found within the genome and between nuclear and mtDNA could be the result of either incomplete lineage sorting or post-divergence gene-flow.

### Divergence time and admixture analysis

In order to differentiate between incomplete lineage sorting and gene-flow, we conducted an independent admixture analysis (using D-statistics) that directly addressed this issue [26] (see Materials and methods; Additional file 4). Overall, we found strong evidence of admixture among species living on Sundaland. Indeed, results of D-statistics (Materials and methods; Additional files 4 and 5) show that species living on Sundaland share a significant excess of derived alleles compared to what would be expected for a simple bifurcating scenario, as displayed in Figure 1B,C. In addition, we found further admixture signatures that involve species living outside of Sundaland. For a detailed discussion of these results, please refer to Additional file 4.

To put the admixture and divergence events in a temporal context, we first estimated molecular divergence times using a relaxed molecular clock as implemented in MCMCtree [27]. In order to account for the uncertainty in fossil dates, we used three separate fossil calibrations to place prior distributions on node age (see Additional file 6 for further discussion and references on the fossil calibrations used in this study). We then selected genomic loci supporting the main topology to obtain the date of original divergence between taxa (Figure 1B), thereby limiting the bias that arises from admixture between species (Additional files 4 and 5).

**Figure 2:**
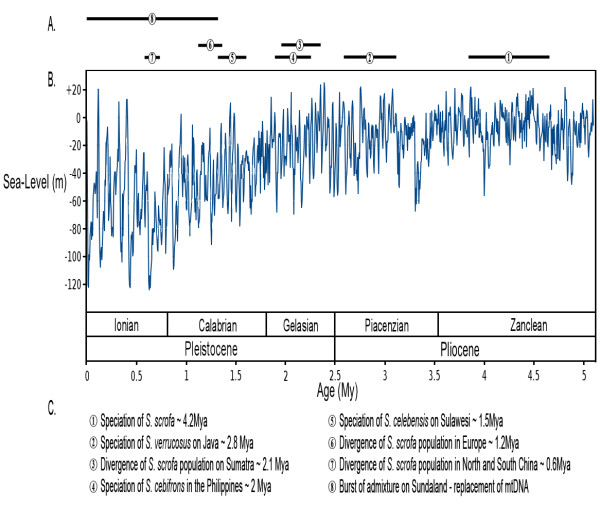
**A eustatic curve adapted from **[18]. **(A) **Each black bar shows 95% confidence interval of each divergence event as inferred from molecular clock analysis (Figure 1B). **(B) **Eustatic curve for the last 5 My. **(C) **Legend of events represented as black bars in (A).

The correlation between the timing of the nodes on the phylogenetic tree and climate models [28] suggested that when global sea levels dropped during cold intervals, the resulting land bridges between islands allowed pigs to disperse across what were once sea barriers (Figures 1A and 2). Warm periods raised sea levels, closed migration routes and isolated populations on individual islands, leading to allopatric speciation. In addition, our admixture analysis revealed the existence of extensive inter-specific gene-flow that likely took place during cold intervals since these periods would have induced parapatric conditions via the connection of previously isolated islands.

### Demographic analysis

**Figure 3:**
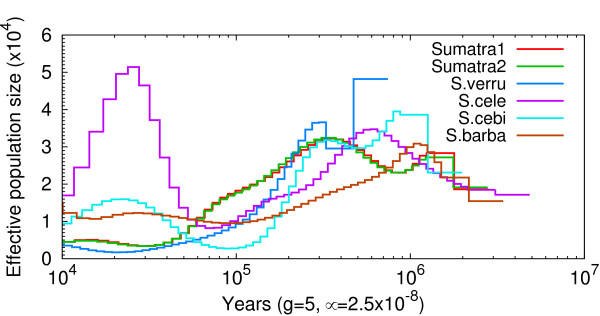
**Population sizes of *Sus *in ISEA inferred from autosomes**. Sumatra1/2 = *S. scrofa *population from Sumatra. S.verru = *S. verrucosus*; S.cele = *S. celebensis*; S.cebi = *S. cebifrons*; S.barba = *S. barbatus*.

We used heterozygous SNP calls for demographic inference in a single individual genome sequence as implemented in PSMC (Materials and methods; Figure 3; Additional file 7). We found that the Pleistocene period led to a bottleneck in both ISEA (Figure 3) and MSEA populations (Additional file 7). These population size declines are consistent with the reduction of temperature observed during this period that would have reduced the overall forest cover in MSEA and ISEA [29,30] (Figure 2). In addition, our results suggest that the populations from ISEA (Figure 3) have undergone a more severe bottleneck than populations of MSEA (Additional file 7).

## Discussion

Our results reveal that, unlike alternative strategies including SNP genotypes (from SNP microarrays), ascertained in a single species or population, that possess inherent biases in between species or population studies [31], whole-genome sequencing (leading to the detection of millions of polymorphisms) allow for phylogenetic relationships and admixture patterns within the genus *Sus *to be confidently resolved. Indeed, when attempting to recapitulate the analysis using the porcine 60K SNP chip [32] (Additional file 8), substantial differences in branch length estimates were found. These discrepancies are due to ascertainment bias demonstrating that a simple SNP array genotyping method, even for multiple individuals, would not have allowed the resolution afforded by a single complete genome. In addition, we show that there is a high degree of phylogenetic discordance across the genome. Such discordance could potentially lead to incorrect conclusions about the relationships between these species if only a subset of these loci were sampled [16]. While phylogenetic incongruence can frustrate taxonomic inference, it has the potential to test for the presence of inter-specific gene-flow. Our data demonstrate that the wealth of information extracted from these genomes allows for a thorough analysis (Additional files 4 and 5) that permits for the temporal reconstruction of the evolutionary history of *Sus *discussed below.

### Evolutionary history of *Sus*

Our divergence time estimates suggest that the initial divergence of the Eurasian wild boar from a clade consisting of other *Sus *species took place during the Zanclean stage at the beginning of the Pliocene (Figure 1B; 5.3 to 3.5 Mya). Though the precise geographic location of this split (either in Sundaland or mainland Southeast Asia) remains unclear, the timing coincides with the divergence between other Sundaic and mainland Asian taxa [10]. The subsequent millions of years (from 3.5 to 2.5 Mya, the Piacenzian stage; Figures 1B and 2) were marked by more intense cold periods that likely facilitated the emergence of a contiguous Sundaland landmass for prolonged periods (Figures 1A and 2). Concomitant drops in sea levels are likely to have allowed the dispersal of the ancestor of *Sus verrucosus *to Java (consistent with the fossil record; Additional file 6). The deep split between *S. verrucosus *and other ISEA *Sus *demonstrates that this endangered species *S. verrucosus *represents a distinct lineage. Such a finding has implications for on-going *ex *and *in situ *conservation programs as it shows that this species represents an evident evolutionarily significant unit that deserves specific conservation strategies.

Our results provide evidence that following the divergence of the *S. verrucosus *lineage, the ancestor of *Sus cebifrons *colonized the Philippines during the first stage of the Pleistocene approximately 2.4 to 1.6 Mya (Gelasian stage; Figures 1B and 2). This date correlates with tectonic activity that led to the isolation of the Philippines from Sundaland even during periods of low sea levels [33]. This same period witnessed the divergence between *S. scrofa *populations on Sumatra and mainland East Asia (Figures 1B and 2). However, it is unclear whether this divergence was the result of migration of *S. scrofa *from ISEA to the mainland or *vice versa*. Moreover, this deep divergence between mainland and ISEA wild boars (*S. scrofa*) supports previous morphological studies that advocated the distinctiveness of these ISEA *S. scrofa *sub-species compared to other MSEA populations [13] (that is, the banded pig *S. scrofa vittatus*).

Our results show that *S. celebensis *colonized Sulawesi, from the west (Borneo), during the latter stage of the Pleistocene (Calabrian; Figures 1B and 2), approximately 1.6 to 0.8 Mya. It appears that this colonization occurred despite evidence that the Makassar Strait separating Sundaland and Sulawesi continued to exist even during periods of lowered sea levels, thus restricting dispersal during the Plio-Pleistocene [34]. Nonetheless, more frequent incidences of lower sea levels during this period [28] (Figure 2) would have reduced the distance between Sundaland and Sulawesi, thereby increasing the likelihood of a successful crossing of the strait. Our phylogenomic analysis implies that populations on Borneo acted as the initial and main source for this dispersal even though the admixture analysis suggest that *S. verrucosus *on Java and *S. cebifrons *in the Philippines later also contributed to the *S. celebensis *gene pool (Additional files 4 and 5). These results may explain the existence of two well-supported but paraphyletic *S. celebensis *mtDNA clades present on Sulawesi [15,25].

While the overseas dispersal of indigenous suids from Java and the Philippines into Sulawesi may have been the result of human-aided translocation, the initial divergence of *S. celebensis *from the Bornean population is too old to have been induced by modern humans. Thus, if overseas dispersal took place between Borneo and Sulawesi, it may also have been possible for pigs to disperse naturally from Java and the Philippines, within the last few million years (for example, by rafting or swimming). Further studies that can date these colonization events from Java and the Philippines into Sulawesi, using multiple genomes from *S. celebensis*, could enable assessments of whether these migrations were in fact the result of human translocation.

The mainland divergence of *S. scrofa *into regionally discrete populations also started during the mid-Pleistocene (Figure 1B). Populations of *S. scrofa *from Asia migrated west approximately 1.2 Mya, reaching Europe around 0.8 Mya as suggested by the first appearance of *S. scrofa *in the fossil record (see Additional file 6 for details). The first divergence between Eastern and Western *S. scrofa*, as timed by our molecular clock analysis (Figure 1B), was likely the result of cooler climate during the Calabrian period that isolated populations in small refugia across Eurasia (Figure 2). Our data indicate that the split between Northern and Southern Chinese *S. scrofa *populations took place during the Ionian stage approximately 0.6 Mya (Figure 1B). This timing correlates with the most significant reduction in global temperature in the Plio-Pleistocene, characterized by long glacial intervals and short interglacial periods, that started approximately 0.8 Mya [35] (Ionian stage; Figures 1B and 2). In this period forests contracted into small refugia, thereby isolating populations across MSEA [10].

### Admixture and mtDNA replacement

Though we have presented the evolutionary history of *Sus *as speciation events resulting from simple bifurcations, D-statistics [26] and simulations challenge this view and suggest numerous instances of diversification and reticulation (Additional files 4 and 5). Our analysis shows that concomitant sea level fluctuations allowed for extensive intra- and inter-specific gene-flow during these periods, both within Sundaland and between Sundaland and MSEA (Figure 1C,D; Additional files 4 and 5). Admixture fractions between Sumatran and Chinese *S. scrofa *subpopulations were higher (9.5 to 11%; Additional file 4) than those between Sumatran *S. scrofa *and other *Sus *species on Sundaland (1.3 to 4.2%; Additional file 4). This finding suggests that, during the Pleistocene, more gene-flow took place between Chinese and Sumatran *S. scrofa *populations than between Sumatran *S. scrofa *populations and other *Sus *species living on Sundaland. The geographic distance between Sumatran and Chinese *S. scrofa *populations is much larger than between Sumatran *S. scrofa *and the other *Sus *species that live on Sundaland (for example, *S. verrucosus *and *Sus barbatus*). Thus, this pattern supports a model of ongoing speciation with gene-flow in which interspecies relatedness is more closely correlated with a history of admixture than with current geographic proximity.

Despite these alternating periods of divergence and homogenization, trees constructed using complete genomes recover the modern species designations. The same is not true of previously published mitochondrial phylogenetic trees of pigs from ISEA and MSEA that were able to distinguish geographically distinct populations of *S. scrofa *in Eurasia, but were unable to recover the monophyly of morphologically distinct species living on Sundaland [15,16,25,36]. This paradox could result from either the limited phylogenetic information present in the short mitochondrial fragments used in previous studies, or from the complex pattern of admixture in Sundaland described above (Figure 1C,D).

Our phylogenetic tree based on near-complete mtDNA genomes (Figure 1E) is consistent with previous studies [15,25], supporting a paraphyletic relationship among non-*S. scrofa *species and a monophyletic clade of Sundaland taxa with short branch lengths. In addition our demographic analysis (Figure 3) shows that species living on Sundaland have undergone a long-term population decline, more extended than on MSEA (Additional file 7), during the Pleistocene. These results suggest that there was a replacement of mitochondrial haplotypes that took place across Sundaland during the latter part of the Pleistocene (1.5 Mya to the present; Additional file 4), after the divergence of *S. celebenisis *(Figure 1B,E; Additional file 4). The mtDNA replacement may have been facilitated by small population sizes (Figure 3). Taxa endemic to the Philippines and Sulawesi, isolated from Sundaland, were not involved in this admixture and harbor highly diverged mtDNA haplotypes of both complete mitochondrial sequences and fragments of the control region [15,25] (Figure 1E). This phenomenon is unlikely to be an exception in pigs and has been recently observed in polar bears [3].

### Human-mediated translocation

Though climate change has had the most dramatic and sustained influence on the speciation history of suids, humans have also affected this process. During the last 40,000 years, humans have actively and passively translocated hundreds of species (as commensals, wild, or domestics) within ISEA, Wallacea and Australasia [11], and the signatures of the resulting admixture between suid lineages are evident in the genomic sequences. In addition, *S. scrofa *is an agriculturally important species that has been independently domesticated at least twice in mainland Eurasia (Near-east and China) [25]. The close relationship between humans and pigs make this species more prone to anthropogenic translocations. Indeed, our admixture analysis revealed the existence of inter-specific gene-flow that involved long distance dispersal across barriers that were unlikely to be the result of natural migration pathways.

Previous morphologic [37] and genetic [15] studies suggested that *S. celebensis *was kept captive and transported by humans from Sulawesi to Timor, Flora, Halmahera and Simeulue (Northwest Sumatra). Admixture analyses support these claims by revealing gene-flow from *S. celebensis *into local *S. scrofa *populations on Sumatra and MSEA. Even during cold periods, Sulawesi and Sundaland were separated by a deep sea channel [34]. Thus, it seems unlikely that populations of *S. celebensis*, from Sulawesi, made it back to isolated islands around Sumatra and MSEA within the last 1.5 My since its divergence from *S. barbatus*. In their totality, these results provide evidence that human translocation of suids took place across the region and was not restricted to islands in close proximity to Sulawesi.

We also detected a strong signature of gene-flow from European *S. scrofa *populations into species in ISEA, consistent with a previous study that identified European mitochondrial haplotypes among populations in ISEA [15]. This gene-flow was most likely the result of human-induced dispersal of European pigs into ISEA within the past few hundred years. Some of these introduced pigs likely became feral and interbred with indigenous species.

While some of the admixture signals detected in this study are unequivocal (that is, admixture within Sundaland, supported by mtDNA and frequent merging of these islands during the Plio-Pleistocene epoch), other signatures, including those involving long distance dispersal, are more difficult to interpret. For example, admixture involving un-sampled or extinct lineages can result in complex site patterns and could influence the results of the D-statistics [26]. For instance, the signal of gene-flow from European *S. scrofa *into species in ISEA could be the result of an admixture from an un-sampled sister lineage, and may not necessarily involve European pigs *per se*. Another limitation of the method can arise from ancestral population subdivision as has been suggested to account for signatures of Neanderthal and human admixture [38]. However, ancestral subdivision is unlikely to affect our analysis because of the evolutionary time frame investigated here (Additional file 4).

### Factors driving and reversing speciation in *Sus*

Our results suggest that Plio-Pleistocene climatic fluctuations had a significant impact on the diversification and homogenization of *Sus *in ISEA and MSEA. Speciation within *Sus *was mainly driven by dispersal across ISEA during the short glacial interval of the late Pliocene and early Pleistocene as suggested by evidence gleaned from other taxa [10,39]. Rapid changes in climate and sea level resulted in population bottlenecks across ISEA (Figure 3). In addition, extensive intra- and inter-specific gene-flow led to instances of mtDNA replacement and a reversal (however temporary) of the speciation process.

### Methodological challenges

Our work demonstrates that the analysis of high-throughput sequencing data provides a powerful tool to investigate speciation history; but is unlikely to be devoid of sequencing errors, especially for low sequence coverage. However, the sequence coverage in our samples (7.5 to 25×) is expected to provide reliable genotype calls [40]. In addition, the major conclusions of this study are not expected to suffer from these biases as these analyses rely on non-singleton sites. Specifically, for a site to be phylogenetically informative the mutation must be shared by at least two taxa and the D-statistic analysis is explicitly designed to be robust to sequencing errors resulting in singletons [26]. Therefore, for a sequencing error to influence our phylogenetic or admixture analysis, it would have to be systematic and have occurred separately in different samples sequenced at different times in different sequencing centers. Thus, making the reasonable assumption that sequencing errors are independent between the samples, the probability of creating enough falsely informative sites to bias these analyses is exceedingly low.

Another limitation of our phylogenetic analysis could stem from recombination. Indeed, due to recombination, each of our genomic bins may represent a mosaic of different evolutionary histories. Nonetheless, theory and simulations suggest that our overall conclusions are relatively insensitive to the effects of recombination [41]. This insensitivity is because, moving along a sequence, different topologies are highly correlated and hence recombination is expected to have small effects over short recombination distances [42].

Lastly, it is important to take results of demographic history with caution. Indeed, while we believe that the general pattern described in Figure 3 is reliable, the magnitude of this bottleneck, in different species, is difficult to interpret. Differences in coverage among our samples likely result in variable power to call heterozygous sites, and could explain at least some of the differences in demographic history between different species.

## Conclusions

The resolution afforded by complete genomes allowed us to infer not only ancient admixture episodes, but also those that took place as a result of more recent human-aided dispersal. Together, these findings provide insights related to the possible response to future climate and anthropogenic disturbances of mammalian taxa within ISEA.

Despite the challenges in building a single phylogeny from entire genome sequences, we were able to obtain a well-resolved tree. In fact, the complexity of whole-genome data allows for a deeper appreciation of the complexities involved in the speciation process. Moreover, the substantial volume of data allows for robust time estimation. These findings reveal the power of multiple complete genomes from closely related species to comprehensively infer their speciation and evolutionary history and to resolve discrepancies between discordant trees constructed using smaller marker sets.

The complete genomes presented here provide compelling evidence that speciation in ISEA suids did not proceed according to a simple bifurcating model. Instead, our data indicate that the process involved numerous periods of both diversification and reticulation amongst several species and is on-going. Extensive inter-specific gene-flow has also been reported in fish [43,44] and birds [45,46]. The resolution afforded by complete genomes reveals that speciation is rarely as simple or linear as our traditional depictions, and that complex patterns of diversification and reticulation are likely the rule and not the exception.

The origin of new species often includes significant time periods during which closely related taxa in the initial stages of diversifying from one another can (and do) produce fertile offspring. The resolution provided by the use of whole genomes allows not only for an assessment of the current and past integrity of species, but also the elucidation of taxa-specific speciation history. Genomics can thus reveal the molecular variability of life on earth, elucidate the process by which it emerged, and inform our attempts to preserve it.

## Materials and methods

### Sequencing, alignment and SNP calling

The samples used in this study were chosen from a larger pool of genotyped individuals (Illumina Porcine SNP60 chip) [32] in each species or population in order to ensure that each was representative of the genetic diversity of their respective species/populations (Additional file 8). DNA was extracted from blood or tissue using the DNeasy blood and tissue kits (Qiagen, Venlo, NL, USA). Quality and quantity were measured with the Qubit 2.0 Fluorometer (Life Technologies, Carlsbad, CA, USA). Libraries of approximately 300 bp fragments were prepared using Illumina paired-end kits (Illumina, San Diego, CA, USA) and sequenced with Illumina GAII or HiSeq (Table S1 in Additional file 1).

Reads were trimmed for three consecutive base pairs with phred quality score equal or below 13, and discarded if they were shorter than 40 bp. We used Mosaik 1.1.0017 with the unique alignment option to align reads to the Swine reference genome (Sscrofa10.2; GenBank GCA_000003025.4; Table S2 in Additional file 1), together with the complete, mtDNA genome of *S. scrofa *(accession: AF486874) for all *Sus *species and the mtDNA genome of *Phacochoerus africanus *(accession: DQ409327) for *P. africanus*. The *S. scrofa *and *P. africanus *mtDNA genomes were aligned using ClustalW [47]. Mapping errors are unlikely to be problematic in this study, as the sequence mismatch to the reference genome was at max 3 to 4% (3 to 4 mismatches per 100 bp read), a distance easily accommodated by short-read local aligners such as Mosaik. Mapped read depth ranged from 7.5 to 24× (Table S1 in Additional file 1), thus providing enough power to call genotype confidently [40]. The resulting BAM files were deposited on the EBI Sequence Read Archive under accession number ERP001813.

We used the pileup format (Samtools [48]) to call genotype at sites covered by at least three reads with minimum base and mapping quality of 20. Additionally, we excluded any clusters of three or more SNPs within 10 bp or any SNP within 3 bp of an indel. We then identified genomic bins of 1 kbp that had an average depth under a maximum threshold (twice genome-wide average coverage) and 90% nucleotide sequence covered, to ensure maximum sequence coverage in every sample and exclude false positive SNPs resulting from copy number variation. These genomic bins were chained if adjacent.

Lastly, we calculated the intersection of the genomic bins previously identified in each individual for further analysis using BedTools [49]. This resulted in an 11 way alignment with maximum sequence coverage and minimum false positive SNP calling in all our samples (approximately 1.1 Gbp; Table S3 in Additional file 1).

We computed the distance to an outgroup (African warthog) in 1 Mbp windows for every *Sus *sample. Thereafter, we computed mean distances of all *Sus *to the outgroup. We obtained recombination rates from [20]. We used Kendall's rank test for correlation analysis as implemented in R.

Because the depth of coverage of mtDNA was highly variable across the different samples (Table S4 in Additional file 1), we applied a different filtering strategy. For each position covered we calculated the effective coverage of each allele as:

(1) $$C\left(j\right)=\Sigma_{i=1}^{depth\left(j\right)}\left(\text{1}-\text{1}{\text{0}}^{-m_{ij}/\text{10}}\right)x\left(\text{1}-\text{1}{\text{0}}^{-q_{ij}/\text{10}}\right)$$

where *m_ij _*and *q_ij _*refer to mapping quality and base quality score for read i at position j [50]. We filtered any sites where the major allele effective coverage did not represent at least 70% of the overall effective coverage at the position.

### Phylogenetic analysis

First, we randomly selected genomic fragments (Table S3 in Additional file 1) of at least 1 kbp to make up 100 unique alignments of 1 Mbp (between 0.99 Mbp and 1.1 Mbp/each). We fitted a GTR+Γ_4_+I model of sequence evolution to each partition (genomic fragment) and ran 100 fast bootstrap replicates for each alignment and a thorough ML search using RAxML 7.1.2 [23]. We constructed a frequency consensus tree using all bootstrap replicates obtained from the 100 unique alignments using Phylip CONSENSE package [51]. These frequencies were then used as support for the species tree (Additional file 2).

To reconstruct the mtDNA tree we used a Bayesian tree reconstruction with 50,000,000 MCMC samples as implemented in MrBayes v3.2 [52]. We fitted a GTR+Γ_4_+I model suggested by AIC criterion as implemented in MrAIC [53]. We assessed the convergence of MCMC samples using TRACER [54]. The resulting phylogenetic tree is presented in Figure 1E.

To assess the robustness of these supermatrices we also applied more formal supertree methods by estimating a ML tree using RAxML with 100 fast bootstrap replicates for each genomic bin of at least 5 kbp (Table S3 in Additional file 1). We used STAR [24] to reconstruct the species tree. Thereafter, we computed the relative frequency for each observed clade (Additional file 3). Relative frequencies correspond to the proportion of each clade in the database of bootstrapped single locus trees.

In order to investigate how recombination affects phylogenetic concordance across the genome we computed the mean pairwise Robison-Foulds distance of trees, using Phylip [51], within 1 Mbp windows. We obtained recombination rates from [20]. We used Kendall's rank test for correlation analysis as implemented in R.

### Molecular clock analyses

We estimated divergence times using an approximate likelihood method as implemented in MCMCtree (PAML v.4), with an independent relaxed-clock and birth-death sampling [27]. To overcome difficulties arising from computational efficiency and admixture, we only used fragments (minimum 5 kbp) that had a good bootstrap support (at least 70% bootstrap support for each node) for the main topology (Additional file 2). Although this is expected to bias estimates of divergence time toward the present, the amount of error is expected to be relatively small considering the deep time scale in this analysis. This resulted in 416 genomic bins and a 4.4 Mbp alignment. We fitted an HKY+Γ_4 _model to each partition (bin) and estimated a mean mutation rate by fitting a strict clock to each fragment setting a root age at 10.5 Mya, as suggested by previous studies [55]. This mean rate was used to adjust the prior on the mutation rate (rgene) modeled by a gamma distribution as G (1,125). Parameters for the birth-death process with species sampling prior (BDS) and sigma^2 ^values were set at 7 5 1 and G (1, 10), respectively. We ran two independent 40,000 (+10,000 burn in) MCMC samples for each combination of fossil calibration (Additional file 6) and assessed the convergence using TRACER [46] (Effective Sample Size [ESS] > 100).

### Demographic analysis

We conducted a demographic analysis using a hidden Markov model approach as implemented in PSMC [56] in our ISEA samples. We generated consensus sequences from bam files using the 'pileup' command in SAMtools. We used the following parameters: T_max _= 20; n = 64 ('4+50*1+4+6'). For plotting the results we used g = 5 and a rate of 2.5 × 10^-8 ^mutations per generation as in humans.

### Admixture analyses

To detect and quantify admixture among taxa we used D-statistics [6,26] that take advantage of the large number of SNPs present in whole genomes. In short, the D-statistics provide a robust test for admixture by assessing the fit of a strictly bifurcating phylogenetic tree. For a triplet of taxa P1, P2 and P3, and an outgroup O, in which the underlying phylogeny is represented by the Newick string (((P1, P2), P3). O), one can compute the number of sites with mutations consistent with incomplete lineage sorting: those where P1 and P3 (BABA) or P2 and P3 (ABBA) share the derived allele (B; assuming ancestral state, A, in the outgroup). Under a null hypothesis of no gene-flow (strict bifurcation), the ratio D = (ABBA - BABA)/(ABBA + BABA) is not expected to be significantly different from 0. This is because ABBA and BABA sites can only be created by coalescences in the common ancestor of P1, P2 and P3 and hence should happen with equal frequency. Alternatively, a significant excess of either ABBA or BABA site patterns is inconsistent with incomplete lineage sorting and provides evidence for a deviation from a phylogenetic tree, suggesting additional population structure or gene-flow.

To compute a standard error and assess the significance of the D-statistics, we used a Weighted Block Jackknife approach. We divided the genome into N blocks and computed the variance of the statistics over the genome N times leaving each block aside and derived a standard error (SE) using the theory of the Jackknife (supplementary online material 15 in [6]). We then computed the D-statistics for every possible combination of species (Additional files 4 and 5) using *P. africanus *as an outgroup. We corrected for multiple testing using a simple Bonferroni correction that involved multiplying our pvalues by the number of D calculation (Additional files 4 and 5). For additional details see Additional file 4.

## Abbreviations

bp: base pair; ISEA: Island Southeast Asia; ML: maximum likelihood; MSEA: Mainland Southeast Asia; mtDNA: mitochondrial DNA; My: millions years; Mya: million years ago; SNP single nucleotide polymorphism.

## Competing interests

The authors declare that they have no competing interests.

## Authors' contributions

MAMG, ALA, LBS, H-JM, OM, GL and LAFF designed the study. LAFF and H-JM carried out sequence alignment and SNP calling. LAFF and JGS analyzed the data with input from OM, HJ-M and MS. RPMAC performed the DNA extraction and library preparation. GS, NL, ALA, EM and LBS provided samples and helped with the design of the study. YP and MB helped with the design of the study and the bioinformatics analyses. LAFF, JGS and GL wrote the manuscript with input from OM, EM, H-JM, ALA, MS and MAMG. All authors read and approved the final manuscript.

## Supplementary Material

Additional file 1 **Tables S1 to S4, with information on sequence data and alignment results**. Click here for file

Additional file 2 **Figure S1, a species cladogram with support from various analyses**. Click here for file

Additional file 3 **Table S5, containing results from clade relative frequency analysis**. Click here for file

Additional file 4 **Text with additional results and discussion for admixture analysis**. Click here for file

Additional file 5 **Table S8, which contains the full results from the D-statistics analysis**. Click here for file

Additional file 6 **Text that contains information about fossil calibration**. Click here for file

Additional file 7 **Figure S3 describing the demographic history of the population from MSEA**. Click here for file

Additional file 8 **Figure S4, a phylogenetic tree constructed using SNPs sequenced with the Illumina Porcine SNP60 array**. Click here for file
