# Supplementary material for: Genome sequencing reveals fine scale diversification and reticulation history during speciation in Sus
Source: Genome Biol. 2013 Sep 26;14(9):R107. doi: 10.1186/gb-2013-14-9-r107 (PMC4053821; doi:10.1186/gb-2013-14-9-r107)
Supplement: Additional file 1 — Tables S1 to S4, with information on sequence data and alignment results. [file gb-2013-14-9-r107-S1.PDF]

Additional file 1 – Next Generation Sequencing summary

Sample	Range	Origin of sample	Sequencing Platform	Coverage
Phacochoerus Africanus	Western and Central Africa	Omaha's Zoo, USA	Illumnia GA II + HiSeq	13 x
Sus barbatus	Borneo, Sumatra and Malaysia	Surabaya Zoo, Indonesia	Illumina GA II	7.5 x
Sus celebensis	Sulawesi	Sulawesi (wild)	Illumina GA II + HiSeq	24 x
Sus cebifrons	Philippines	San Diego Zoo, USA	Illumnia HiSeq	10 x
Sus verrucosus	Java	Surabaya Zoo, Indonesia	Illumina GA II	13 x
Sus scrofa Sumatra 1	Sumatra	North Sumatra (wild)	Illumina GA II	11 x
Sus scrofa Sumatra 2	Sumatra	North Sumatra (wild)	Illumina HiSeq	11 x
Sus scrofa North	North China	North China (wild)	Illumnia HiSeq	10 x
Sus scrofa South China	South China	South China (wild)	Illumnia HiSeq	10 x
Sus scrofa Europe – France	France	South France (Wild)	Illumnia HiSeq	10 x
Sus scrofa Europe – Italy	Italy	North Italy (wild)	Illumina HiSeq	14 x

Table S1: Summary of samples with sequence coverage and origin.

Sample	Read depth	Reads mapped	% Reads mapped	SNPs
Phacochoerus Africanus	13	280,820,422	89.3%	25,722,349
Sus barbatus	7.5	153,300,572	72.4%	12,866,808
Sus celebensis	24	566,208,248	89.3%	18,100,987
Sus cebifrons	10	211,417,574	90.0%	14,182,622
Sus verrucosus	13	304,369,740	88.5%	15,587,942
Sus scrofa Sumatra 1	11	252,939,645	91.4%	10,036,807
Sus scrofa Sumatra 2	11	242,430,585	91.4 %	9,612,052
Sus scrofa North China	10	224,029,430	90.4%	8,255,632
Sus scrofa South China	10	225,292,809	90.8%	9,278,691
Sus scrofa Europe - France	10	201,870,333	90%	4,315,720
Sus scrofa Europe – Italy	14	313,128,878	90.5%	4,565,236

Table S2: Number of reads mapped and filtered SNP against reference, for nuclear DNA. Name in brackets are aliases that are used throughout the supplementary materials.

	Total size	Average size	Proportion of whole genome
All	1,107,272,113 bp	2,621 bp	~ 45 %
Less than 5kbp	614,493,307 bp	1,705 bp	~ 25 %
Over 5kbp less than 10 kbp	325,991,279 bp	6,882 bp	~ 13 %
Over 10kbp	166,787,527 bp	13,676 bp	~ 7 %

Table S3: Summary of fragmented 11way alignment.

Sample	Average Read Depth	Reads Mapped	SNPs
Sus barbatus	5,440	1,059,328	2,644
Sus celebensis	6,822	2,432,553	2,480
Sus cebifrons	7,194	4,576,227	2,657
Sus verrucosus (Sverru)	7,066	2,762,085	2,053
Sus scrofa Sumatra 1	2,392	441,741	2,493
Sus scrofa Sumatra 2	4,216	774,424	2,378
Sus scrofa North China	6,985	1,991,028	1,992
Sus scrofa South China	696	126,176	1,989
Sus scrofa Europe - France	233	40,732	1,907
Sus scrofa Europe – Italy	7,201	2,538,897	1,756

Table S4: mtDNA average read depth, number of reads mapped and filtered SNP against reference.
