# Supplementary material for: Genome sequencing reveals fine scale diversification and reticulation history during speciation in Sus
Source: Genome Biol. 2013 Sep 26;14(9):R107. doi: 10.1186/gb-2013-14-9-r107 (PMC4053821; doi:10.1186/gb-2013-14-9-r107)
Supplement: Additional file 2 — Figure S1, a species cladogram with support from various analyses. [file gb-2013-14-9-r107-S2.PDF]

Additional file 2 – Species Cladogram with support

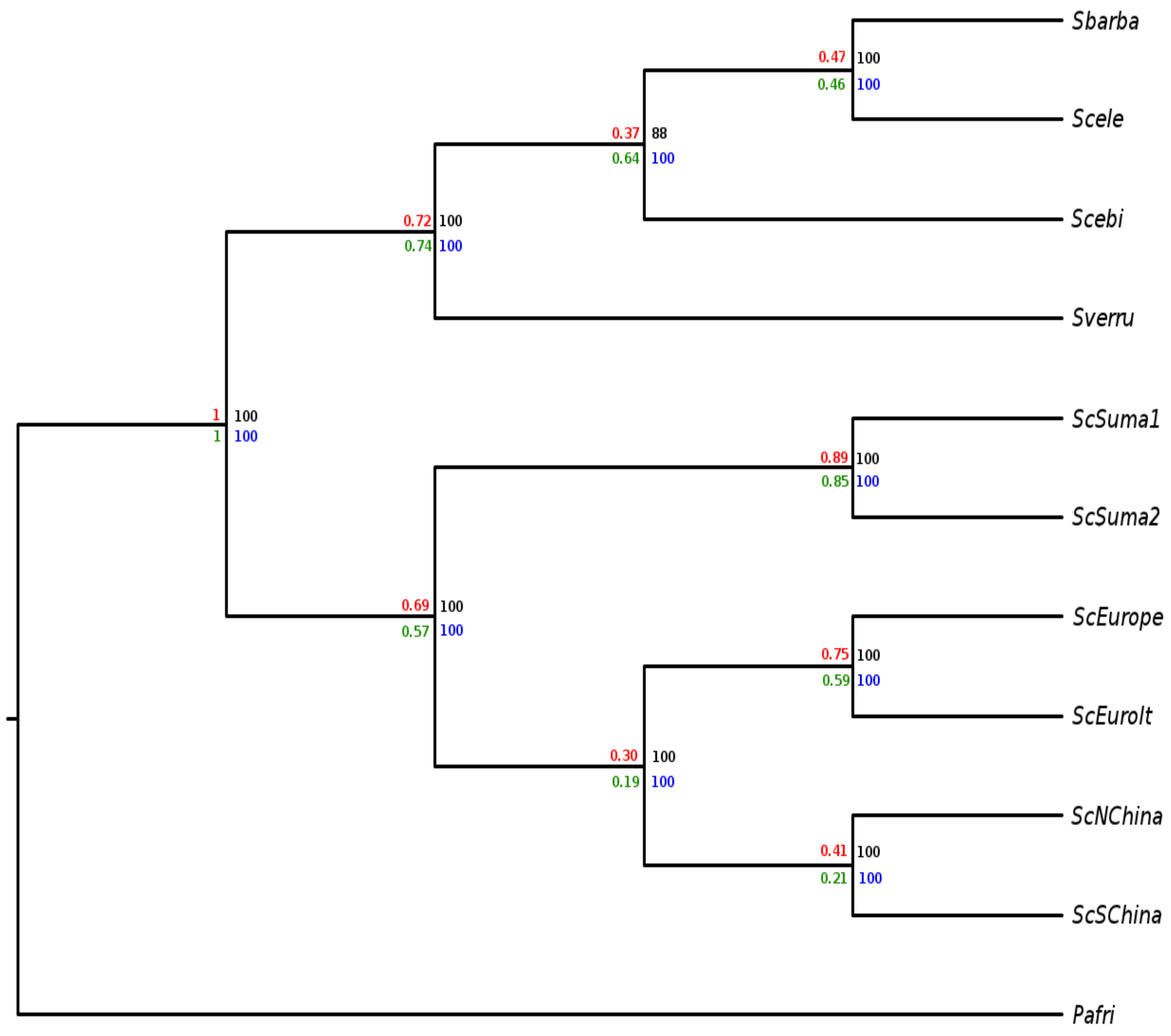

Figure S1: Species cladogram with support. Black color = Support from 1Mbp supermatrix analysis. Blue color = Support from STAR analysis. Red color = concordance factor from autosomal chromosomes. Green color = concordance factor from chromosome X. ScSuma1/2 = *S. scrofa* Sumatra; ScEuroIt = *S. scrofa* Italy; ScEurope = *S. scrofa* Europe; Sbarba = *S. barbatus*; Scebi= *S. celebensis*; Sverru= *S. verrucosus*; ScNChina = *S. scrofa* North China; ScSChina = *S. scrofa* South China.
