# Supplementary material for: Genome sequencing reveals fine scale diversification and reticulation history during speciation in Sus
Source: Genome Biol. 2013 Sep 26;14(9):R107. doi: 10.1186/gb-2013-14-9-r107 (PMC4053821; doi:10.1186/gb-2013-14-9-r107)
Supplement: Additional file 3 — Table S5, containing results from clade relative frequency analysis. [file gb-2013-14-9-r107-S3.PDF]

Additional file 3 – Relative frequency for different clade.

Clade	All chr.	chr. X
ScSuma1,ScSuma2	0.89	0.85
ScEuroIt,ScEurope	0.75	0.59
Sbarba,Scebi,Scele,Sverru	0.72	0.75
ScEuroIt,ScEurope,ScNChina,ScSChina,ScSuma1,ScSuma2	0.69	0.57
Sbarba,Scele	0.47	0.46
ScNChina,ScSChina	0.41	0.20
Sbarba,Scebi,Scele	0.37	0.64
ScEuroIt,ScEurope,ScNChina,ScSChina	0.20	0.55
ScNChina,ScSChina,ScSuma1,ScSuma2	0.18	0.08
Scebi,Scele	0.16	0.32
Scele,Sverru	0.15	0.08
ScSChina,ScSuma1,ScSuma2	0.13	0.48
Scebi,Sverru	0.13	0.07
Sbarba,Scebi	0.12	0.08
ScEuroIt,ScEurope,ScSChina	0.12	0.05
ScEuroIt,ScEurope,ScSuma1,ScSuma2	0.12	0.05
Scebi,Scele,Sverru	0.1	0.15
Sbarba,Sverru	0.09	0.03
Sbarba,ScSuma1,ScSuma2,Scebi,Scele,Sverru	0.07	0.01

Table S5: Relative frequency obtained from autosomal and X chromosome. ScSuma1/2 = *S.*

scrofa Sumatra; ScEuroIt = *S. scrofa* Italy; ScEurope = *S. scrofa* Europe; Sbarba = *S. barbatus*; Scebi= *S. celebensis*; Sverru= *S. verrucosus*; ScNChina = *S. scrofa* North China; ScSChina = *S. scrofa* South China.
