# Supplementary material for: Genome sequencing reveals fine scale diversification and reticulation history during speciation in Sus
Source: Genome Biol. 2013 Sep 26;14(9):R107. doi: 10.1186/gb-2013-14-9-r107 (PMC4053821; doi:10.1186/gb-2013-14-9-r107)
Supplement: Additional file 4 — Text with additional results and discussion for admixture analysis. [file gb-2013-14-9-r107-S4.PDF]

Additional file 4 – Admixture analysis

CONTENTS

1. Detecting admixture in single genome sequences

2. Admixture fraction

3. Confounded D-statistics

4. Interpretation of D-statistics

4.1 Sunda-shelf admixture

4.2 Natural dispersal in and out ISEA

4.3 Natural dispersal into Sulawesi

4.4 Human-mediated admixture

5. Timing admixture

6. References

7. Tables and Figures

1. Detecting admixture in single genome sequences

Several studies have estimated admixture fractions between individuals using a range of methods[1,2]. Programs such as SABER[3] can compute the admixture fraction from one population to another, using High-Density SNP genotypes. However, this method requires multiple samples from the sample population, to estimate Linkage Disequilibrium (LD), identify admixture blocks and estimate time of admixture. This approach is not applicable on single whole-genome sequence. Instead we decided to use D-statistics, originally implemented in the Neanderthal genome paper[2] and formalized by Durand *et al*. (2011)[4]. The D-statistics take advantage of the large number of SNPs present in whole genomes to infer admixture. Assume that we have sequenced one chromosome from 4 different populations P_1, P_2, P_3 and O, where P_1 and P_2 are sister taxa and O is an outgroup. One can compute number of derived alleles that match between P_1 and P_3 (ABBA count) and between P_2 and P_3 (BABA count). Under a null hypothesis of no gene flow between P_3 and either P_2 or P_1 we expect a similar count of

ABBA and BABA patterns to arise from incomplete lineage sorting. Under an alternative scenario of admixture, ABBA counts may be significantly higher than BABA counts (or *vice versa*), which is indicative of gene flow between P_2 and P_3. For a full description of the method please refer to Durand *et al.* (2011). To compute a standard error on the D-statistics we used a Weighted Block Jackknife approach. Briefly, we divided the genome into N blocks and computed the variance of the statistics over the genome N times leaving each block aside and derived a standard error (SE) using the theory of the Jackknife (For full approach see Supplementary Online Material 15 in Green *et al.* 2010). We then computed the D-statistics for every possible combination of species (**Additional file 5**) using *P. Africanus* as an outgroup. We corrected for multiple testing using a simple Bonferroni correction. Simply, we multiplied our p-values by the number of D calculation (360; **Additional file 5**). We tested the influence of different block sizes on the estimation on the SE (**Table S6**). Overall, the SE estimates were slightly higher at 5 and 10Mb blocks size than 2Mb. However, we did not observe significance level higher than 0.01 (after correction) using 2Mb blocks size. Therefore, we used the 2Mb as block size for further analyses. We also assessed the effect of transition and transversion mutations on D estimates. Overall D-statistic computation using transitions or transversions resulted in the same outcome (**Table S7**). We also recomputed the D-statistics at higher coverage to test for the effect of false negative SNP calling. These results were similar to those presented here and show that our method is not sensitive to differences in coverage (data not shown). Lastly, the D-statistics may be sensible to different read length obtained from Illumina sequencing platforms[5]. The authors, found positive correlations between significance level of D-statistics and read-length, however these correlations were not significant. Thus, they noted these differences may lead to borderline significant false positive results. This phenomenon is unlikely to affect our results as read-length is uniform across our samples (raw length = 100bp) and our corrected p-values are always lower than 0.001. Furthermore, the authors also noted that different sequencing platforms (sanger, Illumina and 454) may also influence D-statistics. However, they do not mention if different Illumina technologies (GAII or HiSeq) may also influence. Our data comprise samples sequenced on both systems (**Table S1**). These differences in technologies did not influence our calculation between our Sumatran *S. scrofa* that were sequenced with either GAII or HiSeq (**Table S1&3**). Therefore, we think that this is unlikely to influence our results.

2. Admixture fraction

While the D statistics estimate the fraction of incomplete lineage sorting that is due to admixture, they are not linearly related to the proportion of admixture (Durand et al. 2011). To compute the admixture proportion, we require data from a taxon that is sister to the population that contributed the admixture (it is also possible to get an upper bound on the admixture proportion using two samples from the same population). Consider a scenario where we have samples from the pairs of sister taxa, P_1 and P_2 and P_3 and P_4. If there is a significant D statistic indicating admixture from P_3 into P_2, we can compute the number of sites where P_2 and P_4 share the derived allele, $S(P_1,P_2,P_4)$. We can also compute the number of sites where P_3 and P_4 share the derived allele, $S(P_1,P_3,P_4)$. The portion of the genome of the sample from P_2 that comes from P_3 will then behave as if it were a member of P_3; therefore $S(P1,P_2,P_4)/S(P_1,P_3,P_4) = f$ the admixture proportion. Thus, while Durand *et al.* (2011) showed that $S(P_1,P_2,P_4)/S(P_1,P_3,P_4) = f$ represents an upper bound of the true admixture fraction in the case of simple admixture and constant population size, it is unclear how more complex history effect the estimation of f.

3. Confounded D-statistics

The D-statistics provide evidence of admixture between two populations. However, some of these admixtures might be confounded. For example, suppose we have 4 populations $((P_1, P_2), (P_3, P_4))$, we detect admixture of P_3 with P_2 (P_3/P_2) and P_4 with P_2 (P_4/P_2) (P_3 and P_4 are sister taxa). **Figure S2** shows 5 possible models that can explain such a result. In the first model (**Figure S2 A**) the admixture is completely confounded because it comes from the common ancestor of P_3 and P_4. In the second and third model (**Figure S2 B, C**), the admixtures are partly confounded and involve $P_{3,4}$ and P_3 or P_4 only. In the fourth model (**Figure S2 D**) the admixtures independently involve P_3 and P_4. Finally, in the fifth model (**Figure S2 E**) there are 3 admixtures event that are partly confounded and involve both P_3, P_4 but also $P_{3,4}$. One way to distinguish between these models is to compare the value of D statistics. Durand et al. (2011) showed that the value of D increases with the difference between time of admixture and time of divergence of the 3 taxa involved in the D calculation. In the case described here $D_1(P_1,P_2,P_3)$ tends to 1 as $t_{p1,2,3,4} - t_{GF1}$ becomes large (**Figure S2**). Moreover, D also increases with the admixture fraction f. Because, in this example, $t_{p1,2,3,4}$ is constant across D calculations, a significant increase of D_1 compared to D_2 is the result of, t_{GF1} being smaller than t_{GF2} and t_{GF3} such as $t_{p1,2,3,4} - t_{GF1} > t_{p1,2,3,4} - t_{GF2}$ and

$t_{p1,2,3,4} - t_{GF1} > t_{p1,2,3,4} - t_{GF3}$ and/or due to a higher admixture fraction such as, $f_1 > f_2$. Therefore, there must be a more recent admixture event and/or higher admixture fraction between P_3/P_2 than P_4/P_2 which in turn indicates that the admixture P_3/P_2 is at least partly independent from $P_{3,4}/P_2$ and P_4/P_2. This rationale permits the rejection of models 1 and 3 (**Figure S2 A, D**). We assessed if a pair of D value were significantly different using a Z-test. Briefly, we found the difference between D-statistics of interest and used the sum of the Jackknife variance estimates as an estimate of the variance of this quantity. We then assessed if the difference was significantly different from 0. However, this test does not allow us to distinguish between models 2, 4 and 5 (**Figure S2 B,D,E**). Because we can show that there must be an independent admixture event P_3/P_2 it does not mean that we can rule out the possibility of independent admixture event P_4/P_2 or a confounded event $P_{3,4}/P_2$. To distinguish between these models, one can examine the overlap in sites that support the two D statistics. In the case of confounded admixture, there should be substantial overlap between sites that support a non-zero D-statistic. However, it is unclear how many overlapping ABBA or BABA can be expected under these models, as different population processes may influence their counts. Further studies should concentrate on deriving the number of expected overlapping ABBA and BABA under these three different models.

4. Interpretation of D-statistics

4.1 Sunda-shelf admixture

We detected admixture from Sumatran *S. scrofa* into other species living in the Sunda-shelf. The D-statistics reveal an excess of shared derived lineages between *S. barbatus* and Sumatran *S. scrofa* compared to both *S. cebifrons* and *S. celebensis* (D= 0.0813+-0.0042 ; D= 0.0795+-0.0042). This signal seems to be stronger in *S. verrucosus* (D= 0.1681+-0.0108; D = 0.1696+-0.0079). Moreover, this admixture was also detectable using *S. barbatus* and *S. verrucosus*, where Sumatran samples share more derived alleles with the later (D= 0.1003+-0.0095). This pattern is consistent with the clustering of *S. verrucosus* and Sumtaran *scrofa* in the phylogenetic tree derived from complete mtDNA sequences(**Figure S2**).

This admixture pattern in the Sunda-shelf appears to be bidirectional. We found that derived alleles observed in *S. barbatus* matched more often Sumatran *S. scrofa* than *S. scrofa* from South China, North China and Europe

(D=0.2000+-0.0084; D=0.2016+-0.0086; D=0.2119+-0.0089). The same pattern was observed using *S. verrucosus* as admixting species(D=0.2996+-0.0120; D=0.3028+-0.0123 ;D=0.3311+-0.0116). Finally we found that *S. verrucosus* shares more derived lineages with *S. barbatus* than with *S. cebifrons* (D=0.1648+-0.0042). These findings show that admixture within the Sunda-shelf did not only involve gene flow between *S. scrofa* and non-*scrofa* species, but rather involved all species living on the Sunda-shelf. Thus, these results reinforce the conclusion that inter-specific gene flow resulted in mtDNA replacement in Sundaland and resulted in discordant phylogenetic signal between mtDNA and autosomal chromosomes.

Because we had two individuals from the *S. scrofa* Sumatra population we were able to obtain an upper bound of the admixture fraction from this population into Suda-shelf species.

- Admixture fraction of Sumatran into *S. verrucosus*:

$$f_{ScSuma1,Sverru}=\frac{S(Scebi,Sverru,ScSuma1)}{S(Scebi,ScSuma2,ScSuma1)}=0.042$$

$$f_{ScSuma2,Sverru}=\frac{S(Scebi,Sverru,ScSuma2)}{S(Scebi,ScSuma1,ScSuma2)}=0.040$$

- Admixture fraction of Sumatran into *S. barbatus*:

$$f_{ScSuma1,Sbarba}=\frac{S(Scebi,Sbarba,ScSuma1)}{S(Scebi,ScSuma2,ScSuma1)}=0.016$$

$$f_{ScSuma1,Sbarba}=\frac{S(Scebi,Sbarba,ScSuma1)}{S(Scebi,ScSuma1,ScSuma2)}=0.016$$

$$f_{ScSuma1,Sbarba}=\frac{S(Scele,Sbarba,ScSuma1)}{S(Scele,ScSuma2,ScSuma1)}=0.013$$

$$f_{ScSuma2,Sbarba}=\frac{S(Scele,Sbarba,ScSuma2)}{S(Scele,ScSuma1,ScSuma2)}=0.013$$

We did not attempt to compute the admixture fraction from Sumatran *S. scrofa* into *S. verrucosus* using *S. celebensis* as non-admixting, because we found clear evidence of admixture between *S. verrucosus* and *S. celebensis*, which would bias the calculation (5.4.3). *S. barbatus* shows a higher admixture fraction (from Sumatran *S. scrofa*) when using *S. cebifrons* than *S. celebensis*. This is expected as *S. celebensis* is more closely related to *S. barbatus* than *S. cebifrons*. Thus, this result suggest that some admixture between Sumatran S.

scrofa and S. barbatus may have taken place before the divergence of *S. barbatus* and *S. celebensis* (admixture into their common ancestor). However, these admixture fractions were very close (0.16 versus 0.13). This result suggests that most of the admixture from *S. scrofa* Sumatra into *S. barbatus* took place after the divergence of *S. barbatus* and *S. celebensis* .

We also found evidence of admixture from *S. cebifrons* into Sumatran *S. scrofa*. This observation can be the result of two possibilities. On one hand, independent admixtures from each both *S. barbatus* and *S. cebifrons* into *S. scrofa* Sumatra could explain this observation. On the other hand, gene-flow from their common ancestor could also explain this result. The latter hypothesis seems more plausible as *S. cebifrons* had no means of dispersal into the Sundaland after its divergence from *S. barbatus*. In addition, we found that, approximately 70% of the sites supporting an admixture from *S. cebifrons* into Sumatran *S. scrofa* (derived state in *S. cebifrons* and *S. scrofa* Sumatra and ancestral state in other *S. scrofa* populations) overlapped with sites supporting admixture from *S. barbatus* into Sumatran *S. scrofa*, suggesting admixture from the common ancestor of *S. barbatus* and *S. cebifrons*. Moreover, D (into Sumatran *S. scrofa*) was significantly higher using *S. barbatus* than *S. cebifrons* as admixting taxa ($p < 0.01$). Thus, we interpret this result as a signal for admixtures into *S. scrofa* Sumatra, from the common ancestor of S. barbatus and S. cebifrons and an additional admixture from *S. barbatus* alone. However, although we cannot rule out the possibility of an additional independent event of admixture from *S. cebifrons* into the Sumatran population of *S. scrofa*, this scenario seems unlikely, as *S. cebifrons* had no mean of dispersal to Sumatra.

In addition to the signals of admixture described above, we find an excess of incomplete lineage sorting between the *S. scrofa* populations (Sumatra and MSEA populations) and the other *Sus* species. For example, we identified admixture between *S. barbatus* and Sumatran *S. scrofa* based on a D statistic of 0.2 (D(ScSChina, ScSuma1, Sbarba). This indicates that 20% of the incomplete lineage sorting between *S. barbatus and S. scrofa* is due to admixture. However, because of the deep divergence between *S. scrofa* and the non-*scrofa* species, very little incomplete lineage sorting is expected. Thus, D statistic should be close to 1. Using simulations, we have seen that the observed D statistic is not possible without additional admixture between the ancestor *S. scrofa* and the ancestor of the non-*scrofa* species (J. Schraiber, unpublished observation). Thus, our results suggest continuous inter-specific gene-flow among population of the Sunda-Shelf throughout the Plio-Pleistocene epoch.

4.2 Natural dispersal in and out ISEA

The D-statistics revealed an excess of derived lineage shared between the *S. scrofa* Sumatran population and both South and North Chinese populations when comparing to the European population (D=0.1803+-0.0039; D=0.1938+-0.0036). Moreover, the D-statistics also support more admixture from Sumtaran *S. scrofa* into South Chinese population than into North Chinese population (D=0.0340+-0.0031). We interpret this pattern as isolation by distance, as Sumatra is closer to South China. These results show that admixture out of ISEA happened repeatedly before and after the divergence of North and South Chinese populations.

The admixture from ISEA into MSEA is not only restricted to within *S. scrofa*. We found signals of admixture from *S. barbatus* into both North and South Chinese *scrofa* compared to European population (D=0.0319+-0.0029; D=0.0339+-0.0029). This can be also found from *S. verrucosus*, (D=0.0654+-0.0035; D=0.0681+-0.0032), *S. cebifrons* (D=0.0354+-0.0035; D=0.0414+-0.0033) and *S. celebensis* (D=0.1029+-0.0030; D=0.1100+-0.0030).

We calculated the admixture of Sumatran population into MSEA:

- Admixture fraction into North Chinese:

$$f_{ScSuma1,ScNChina} = \frac{S(ScEurope,ScNchina,ScSuma1)}{S(ScEurope,ScSuma2,ScSuma1)} = 0.096$$

$$f_{ScSuma2,ScNChina} = \frac{S(ScEurope,ScNchina,ScSuma2)}{S(ScEurope,ScSuma1,ScSuma2)} = 0.095$$

- Admixture fraction into South Chinese:

$$f_{ScSuma1,ScSChina} = \frac{S(ScEurope,ScSchina,ScSuma1)}{S(ScEurope,ScSuma2,ScSuma1)} = 0.110$$

$$f_{ScSuma2,ScSChina} = \frac{S(ScEurope,ScSchina,ScSuma2)}{S(ScEurope,ScSuma1,ScSuma2)} = 0.109$$

$$f_{ScSuma1,ScSChina} = \frac{S(ScNChina,ScSchina,ScSuma1)}{S(ScNChina,ScSuma2,ScSuma1)} = 0.016$$

$$f_{ScSuma2,ScSChina}=\frac{S(ScNChina,ScSchina,ScSuma2)}{S(ScNChina,ScSuma1,ScSuma2)}=0.015$$

These results suggest a higher admixture fraction among *S. scrofa* populations than among Sunda-Shelf populations (see section 5.4.1). Moreover, the admixture fractions reveal that most of admixture out of ISEA happened before the divergence between North and South Chinese populations (as these fractions are very close [0.95 vs. 0.11]).

Our results also suggest admixtures event from all MSEA *S. scrofa* into all ISEA species except *S. celebensis*. Counter intuitively, this pattern is stronger from European, rather than from South and North Chinese populations (**Additional file 5**). Two models could explain this result. Under the first model, only one admixture event took place from European pigs due to human translocation and the signal is present in the Chinese population because of their relatedness. Alternatively, admixture happened before and after the divergence of *S. scrofa* populations on the mainland due to natural and human mediated migrations. Because we can show that there are migrations events from ISEA into the mainland (see section 5.2.3) we hypothesize that at least part of the admixture found from *S. scrofa* into the Sunda-shelf is due to a natural process that took place before and probably after the divergence of *S. scrofa* on the mainland. Moreover, we can show that there is more admixture from European than Chinese *S. scrofa* into ISEA species suggesting a distinct migration from Europe into ISEA which would be difficult to reconcile with natural migration (see section 5.2.4). It is also possible that part of the admixture from Chinese *scrofa* into ISEA species is due to Human-mediated dispersal of pigs (see section 5.2.4).
We calculated the admixture proportion from mainland *S. scrofa* into ISEA species due to natural dispersal under this model:

$$f_{Mainland,Sbarba}=\frac{S(Scele,Sbarba,Mainland)}{S(Scele,ScEurope,China)}=0.041$$

$$f_{Mainland,Sverru}=\frac{S(Scele,Sverru,Mainland)}{S(Scele,ScEurope,China)}=0.040$$

$$f_{Mainland,Scebi}=\frac{S(Scele,Scebi,Mainland)}{S(Scele,ScEurope,China)}=0.040$$

where Mainland represents the shared SNP between North, South China and Europe *S. scrofa* that supports these admixtures.

and China represents only the shared SNP between North and South China *S. scrofa.* This result supports the view that most of the admixture between continental Eurasia and the Sunda-shelf is due to natural migrations as most of the admixture from MSEA into ISEA seems to be confounded in the different MSEA S. scrofa.

Together these results show that natural migration from ISEA to MSEA and *vice-versa* took place throughout the mid / late Pleistocene. Thus, because we have no taxa that diverged prior to this period on the mainland, we cannot infer natural migration out ISEA during the late Pliocene and early Pleistocene. However, this is likely to be the case.

4.3 Natural dispersal into Sulawesi

Besides the signal for admixture between *S. verrucosus* and *S. barbatus*, we found evidence for admixture between *S. verrucosus* and *S. celebensis*. The D-statistics supporting an admixture of *S. verrucosus* with *S. barbatus* is significantly lower than the value supporting an admixture with *S. celebensis* (using *S. cebifrons* as non introgressed; D=0.1650+-0.0042; D=0.2595+-0.0040; $p < 0.01$). Only two scenarios can explain such a result: a higher admixture fraction or more recent admixture from *S. verrucosus* into *S. celebensis* than into *S. barbatus* (see section 3). It is impossible, with our data, to distinguish between these hypotheses. However, there was a strong signal of admixture between *S. verrucosus* and *S. celebensis* using *S. barbatus* as a putatively non-introgressed species (D=0.1134+-0.0030). This result strongly supports the idea that *S. verrucosus* admixed with *S. celebensis* after its divergence from *S. barbatus*. Admixture into Sulawesi was also found from *S. cebifrons* (D(Scele,Sbarba,Scebi) = 0.0682 +- 0.0029). This finding shows that both *S. cebifrons* and *S. verrucosus* contributed to *S. celebensis*' gene pool.

4.4 Human-mediated admixture

The admixture found from MSEA into *S. cebifrons* in the Philippines can partly be explained by natural dispersal on the 'Sunda-shelf' before its divergence with *S. barbatus* and *S. celebensis*. However, we found that only 45% and 39% of SNP are shared between admixture of MSEA in *S. barbatus* and *S. cebifrons*, respectively. Therefore, we believe that these were, at least, partly independent. Moreover, North and South Chinese derived

lineages are found more often in *S. cebifrons* than *S. celebensis*. Because *S. celebensis* is more closely related to *S. barbatus* than *S. cebifrons* it is unlikely that these observations were the result of an admixture from MSEA into the common ancestor of *S. cebifrons* and *S. barbatus*. In addition, we know that The Philippines have been completely separated from the Sundaland and MSEA during the latter part of the Pleistocene. Together, these results hint at a human mediated dispersal from MSEA into the Philippines. Such a Human-mediated dispersal of pigs may also have happened throughout ISEA. Again, because we can show that there are natural dispersals out of ISEA we assume that at least part of the admixture MSEA to ISEA is due to natural process. However, although it is not possible, with our data, to reach conclusion on the possibility of human-mediated dispersal, of *S. scrofa* of Asian origin in the rest of ISEA (particularly the Sunda-shelf), this hypothesis seems likely if it happened in Philippines. Further studies, with multiple individuals in which admixture blocks can be identified may provide an answer to this question. We took these results with caution because we could not infer a significant excess of derived lineage shared between *S. cebifrons* with either North or South Chinese populations. This could be due to a power limitation as these two populations are very closely related, or simply because the admixture fraction was so small. However, we would expect a significant difference between North and South Chinese if this admixture was human-mediated as these populations would have diverged long before any humans reached the region. Therefore we could not conclude if this admixture was human-mediated or natural.

The admixture from *S. celebensis*, into Sumatran and Chinese *scrofa* seems to be independent from admixture by *S. barbatus* ($p < 0.01$; see section 5.4.1). This is difficult to reconcile with natural dispersal (see section 5.2.4). Previous studies have already found evidence for human mediated dispersal of *S. celebensis* to Flores and Timor[6,7]. Our analysis suggests that this translocation was probably more generalised to the whole Southeast Asian region rather than restricted to only Timor and Flores.

The D-statistics revealed a distinct admixture from European pigs into ISEA species (5.2.3). Moreover, European mtDNA haplotypes were found in domestic pigs in the Philippines7. Together these results support an admixture event from European pigs into ISEA, which is consistent with the idea that Europeans brought pigs to this area during the past few hundred years.

Furthermore, we found that the number of overlapping sites supporting an admixture between Chinese *S. scrofa*

and *S. barbatus* and European *S. scrofa* and *S. barbatus* was lower in the latter comparison (217,801 vs 284,787). Simulations show that this is consistent with an admixture from the common ancestor of MSEA S. scrofra and an additional burst of admixture from European *S. scrofa* into *S. barbatus*.

5. Timing admixture

Because we cannot estimate the extent of LD in our different populations, we could not time directly admixture events. Further studies, using multiple individuals from different populations will provide the means to identify admixture blocks and assess the age of the admixture signals identified in this study.

6. References

1. vonHoldt, B.M. *et al.* A genome-wide perspective on the evolutionary history of enigmatic wolf-like canids. *Genome research* 21, 1294-305 (2011).
2. Green, R.E. *et al.* A draft sequence of the Neandertal genome. *Science* 328, 710-22 (2010).
3. Tang, H., Coram, M., Wang, P., Zhu, X. & Risch, N. Reconstructing genetic ancestry blocks in admixed individuals. *American journal of human genetics* 79, 1-12 (2006).
4. Durand, E.Y., Patterson, N., Reich, D. & Slatkin, M. Testing for ancient admixture between closely related populations. *Molecular biology and evolution* 28, 2239-52 (2011).
5. Prüfer, K. *et al.* The bonobo genome compared with the chimpanzee and human genomes. *Nature*, in press.
6. Groves, C.P. Of mice and men and pigs in the Indo-Australian Archipelago. *Canberra Anthropology* 7, 1-19 (1984).
7. Larson, G. *et al.* Phylogeny and ancient DNA of Sus provides insights into neolithic expansion in Island Southeast Asia and Oceania. *Proceedings of the National Academy of Sciences of the United States of America* 104, 4834-9 (2007).

7. Tables and Figures

P_1, P_2, P_3	2Mbp, D +- SE	5Mbp, D +- SE	10Mbp, D +- SE
ScNChina, ScSuma1, Sbarba	0.2016 +- 0.0086	0.2016 +- 0.0118	0.2016 +- 0.0136
Sbarba, Scele, Sverru	0.1133 +- 0.0030	0.1133 + 0.0033	0.1133 + 0.0033
Sbarba, Scele, ScEurope	-0.2018 +- 0.0036	-0.2018 +- 0.0043	-0.2018 +- 0.0050
ScNChina, ScSuma1, Scele	0.2340 +- 0.0090	0.2340 +- 0.0122	0.2340 +- 0.0140

Table S6: Examples of the influence of different block size on SE of D-statistics. ScSuma1/2 = *S. scrofa* Sumatra; ScEuroIt = *S. scrofa* Italy; ScEurope = *S. scrofa* Europe; Sbarba = *S.*

barbatus; Scebi= *S. celebensis*; Sverru= *S. verrucosus*; ScNChina = *S. scrofa* North China; ScSChina = *S. scrofa* South China.

P_1, P_2, P_3	Ti, D+-SE	n. ABBA / BABA	Tv, D+-SE	n. ABBA / BABA
ScNChina, ScSuma1, Sbarba	0.2306 +-0.0102	350,113/ 237,240	0.1922 +- 0.0087	118,616 / 74,154
Sbarba, Scele, Sverru	0.1287 +- 0.0041	336,825 / 270,954	0.1083 +- 0.0030	116,775 / 90,130
Sbarba, Scele, ScEurope	-0.2377+- 0.0054	147,174 / 216,285	-0.1912 +- 0.0037	41,459 / 67,316
ScNChina, ScSuma1, Scele	0.2741 +- 0.0105	387,887 / 243,560	0.2285 +- 0.0090	132,497 / 75,486

Table S7: Examples of the influence of Transvertion (Tv) and Transitions (Ti) on D-statistics. ScSuma1/2 = S. scrofa Sumatra; ScEuroIt = S. scrofa Italy; ScEurope = S. scrofa Europe; Sbarba = S. barbatus; Scebi= S. celebensis; Sverru= S. verrucosus; ScNChina = S. scrofa North China; ScSChina = S. scrofa South China.

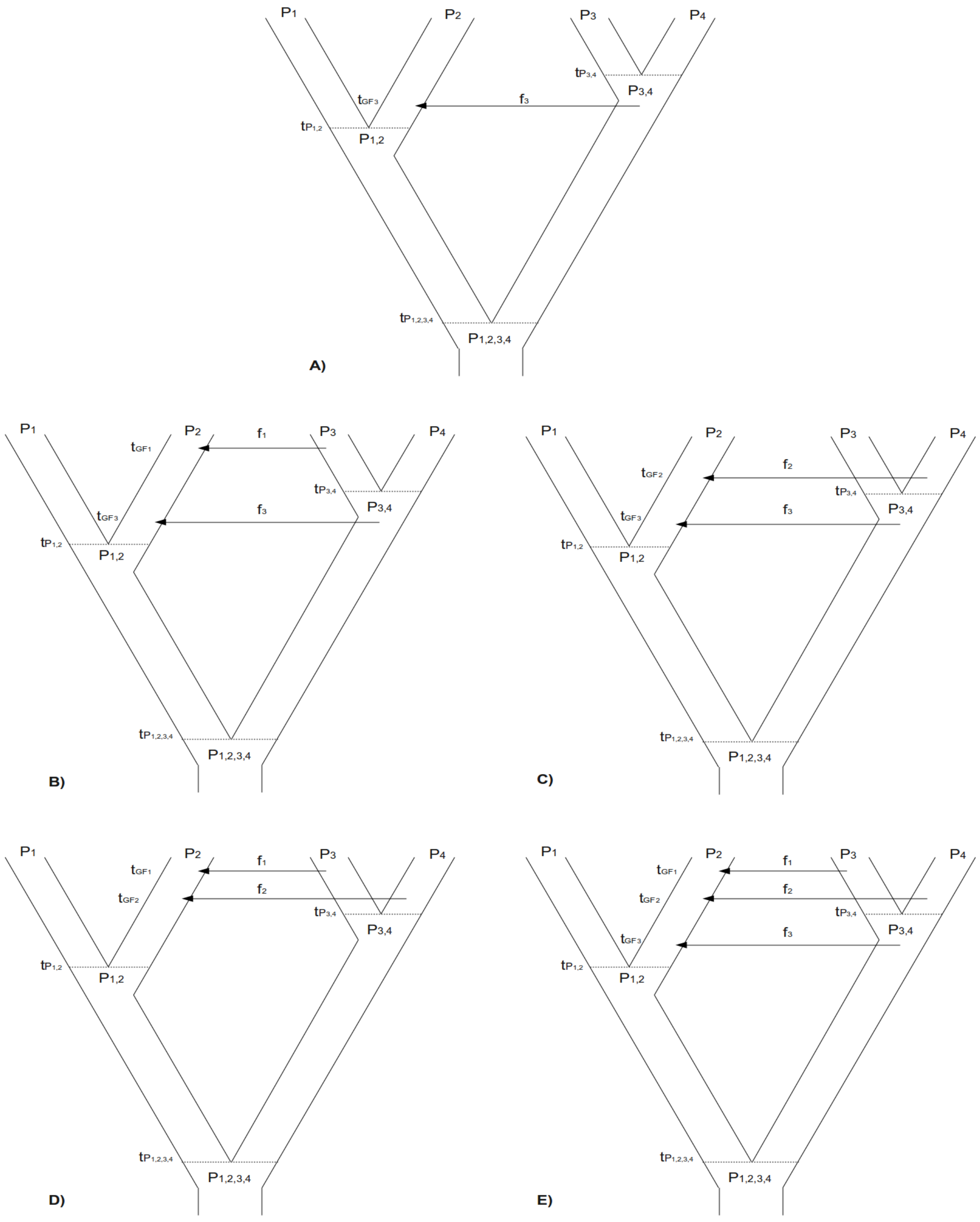

Figure S2: Examples of complex models of admixture, resulting in confounded D-statistics, involving 4 taxa P_1, P_2, P_3 and P_4.
