# Supplementary material for: Genome sequencing reveals fine scale diversification and reticulation history during speciation in Sus
Source: Genome Biol. 2013 Sep 26;14(9):R107. doi: 10.1186/gb-2013-14-9-r107 (PMC4053821; doi:10.1186/gb-2013-14-9-r107)
Supplement: Additional file 5 — Table S8, which contains the full results from the D-statistics analysis. [file gb-2013-14-9-r107-S5.PDF]

P1 P2 P3	ABBA BABA	D+-SE	Interpretation
Scebi ScEuroIt Sbarba	1764675 288263	-0.7192+-0.0029 **	Supports phylogeny
Scebi ScSuma1 Sbarba	1643761 334567	-0.6618+-0.0057 **	Supports phylogeny
Scele Scebi Sbarba	852061 349644	-0.4181+-0.0032 **	Supports phylogeny
Scele ScEuroIt Sbarba	2371394 283627	-0.7863+-0.0023 **	Supports phylogeny
Scele ScSuma1 Sbarba	2191337 283286	-0.7710+-0.0035 **	Supports phylogeny
ScEuroIt ScSuma1 Sbarba	329486 508022	0.2132+-0.0089 **	Admixture from S. barbatus into S. scorfa Sumatra
ScEurope Scebi Sbarba	287356 1763462	0.7198+-0.0029 **	Supports phylogeny
ScEurope Scele Sbarba	284167 2371752	0.7860+-0.0023 **	Supports phylogeny
ScEurope ScEuroIt Sbarba	107496 105864	-0.0076+-0.0042 NS	Non significant
ScEurope ScSuma1 Sbarba	328893 505782	0.2119+-0.0089 **	Admixture from S. barbatus into S. scorfa Sumatra
ScNChina Scebi Sbarba	293298 1752231	0.7132+-0.0033 **	Supports phylogeny
ScNChina Scele Sbarba	270965 2337187	0.7922+-0.0024 **	Supports phylogeny
ScNChina ScEuroIt Sbarba	320981 299601	-0.0345+-0.0029 **	Admixture from S. barbatus into S. scrofa N.China
ScNChina ScEurope Sbarba	317184 297600	-0.0319+-0.0029 **	Admixture from S. barbatus into S. scrofa N.China
ScNChina ScSChina Sbarba	238241 240361	0.0044+-0.0026 NS	Non significant
ScNChina ScSuma1 Sbarba	311559 468912	0.2016+-0.0086 **	Admixture from S. barbatus into S. scorfa Sumatra
ScNChina ScSuma2 Sbarba	306450 461339	0.2017+-0.0086 **	Admixture from S. barbatus into S. scorfa Sumatra
ScNChina Sverru Sbarba	322678 1763198	0.6906+-0.0020 **	Supports phylogeny
ScSChina Scebi Sbarba	294408 1749628	0.7119+-0.0034 **	Supports phylogeny
ScSChina Scele Sbarba	269768 2331138	0.7926+-0.0024 **	Supports phylogeny
ScSChina ScEuroIt Sbarba	340024 316186	-0.0363+-0.0029 **	Admixture from S. barbatus into S. scrofa S.China
ScSChina ScEurope Sbarba	335976 313943	-0.0339+-0.0029 **	Admixture from S. barbatus into S. scrofa S.China
ScSChina ScSuma1 Sbarba	310454 465663	0.2000+-0.0084 **	Admixture from S. barbatus into S. scorfa Sumatra
ScSChina ScSuma2 Sbarba	305718 458253	0.1997+-0.0083 **	Admixture from S. barbatus into S. scorfa Sumatra
ScSuma2 Scebi Sbarba	332066 1638846	0.6630+-0.0056 **	Supports phylogeny
ScSuma2 Scele Sbarba	281842 2186305	0.7716+-0.0035 **	Supports phylogeny
ScSuma2 ScEuroIt Sbarba	500835 324895	-0.2131+-0.0088 **	Admixture from S. barbatus into S. scorfa Sumatra
ScSuma2 ScEurope Sbarba	497933 323509	-0.2123+-0.0089 **	Admixture from S. barbatus into S. scorfa Sumatra
ScSuma2 ScSuma1 Sbarba	100792 101679	0.0044+-0.0115 NS	Non significant
Sverru Scebi Sbarba	560202 649047	0.0735+-0.0088 **	Supports phylogeny
Sverru Scele Sbarba	361747 975125	0.4588+-0.0073 **	Supports phylogeny
Sverru ScEuroIt Sbarba	1785476 323488	-0.6932+-0.0019 **	Supports phylogeny
Sverru ScEurope Sbarba	1784516 323386	-0.6932+-0.0019 **	Supports phylogeny
Sverru ScSChina Sbarba	1759683 323565	-0.6894+-0.0020 **	Supports phylogeny
Sverru ScSuma1 Sbarba	1605847 323275	-0.6648+-0.0028 **	Supports phylogeny
Sverru ScSuma2 Sbarba	1602508 322448	-0.6650+-0.0028 **	Supports phylogeny
Sbarba Scele Scebi	349503 400674	0.0682+-0.0029 **	Admixture from S. cebifrons into S. celebensis
Sbarba ScEuroIt Scebi	1764675 271407	-0.7334+-0.0032 **	Supports phylogeny
Sbarba ScEurope Scebi	1763477 271593	-0.7331+-0.0032 **	Supports phylogeny
Sbarba ScSuma1 Scebi	1643761 284251	-0.7051+-0.0043 **	Supports phylogeny
Scele ScEuroIt Scebi	2013399 320389	-0.7254+-0.0031 **	Supports phylogeny
Scele ScSuma1 Scebi	1852602 315003	-0.7094+-0.0040 **	Supports phylogeny
ScEuroIt ScSuma1 Scebi	327859 479245	0.1876+-0.0070 **	Admixture from S. cebifrons into S. scorfa Sumatra
ScEurope Scele Scebi	321672 2013778	0.7245+-0.0031 **	Supports phylogeny
ScEurope ScEuroIt Scebi	107568 104792	-0.0131+-0.0049 NS	Non significant
ScEurope ScSuma1 Scebi	328286 476809	0.1845+-0.0071 **	Admixture from S. cebifrons into S. scorfa Sumatra
ScNChina Sbarba Scebi	274435 1752195	0.7292+-0.0034 **	Supports phylogeny
ScNChina Scele Scebi	313216 1981323	0.7270+-0.0033 **	Supports phylogeny

ScNChina ScEuroIt Scebi	319583 294770	-0.0404+-0.0031 **	Admixture from S. cebifrons into S. scrofa N. China
ScNChina ScEurope Scebi	315215 293652	-0.0354+-0.0035 **	Admixture from S. cebifrons into S. scrofa N. China
ScNChina ScSChina Scebi	236080 239956	0.0081+-0.0026 NS	Non significant
ScNChina ScSuma1 Scebi	313220 441482	0.1700+-0.0070 **	Admixture from S. cebifrons into S. scorfa Sumatra
ScNChina ScSuma2 Scebi	307940 437841	0.1742+-0.0071 **	Admixture from S. cebifrons into S. scorfa Sumatra
ScNChina Sverru Scebi	326477 1655800	0.6706+-0.0028 **	Supports phylogeny
ScSChina Sbarba Scebi	275722 1749586	0.7277+-0.0034 **	Supports phylogeny
ScSChina Scele Scebi	313035 1975357	0.7264+-0.0033 **	Supports phylogeny
ScSChina ScEuroIt Scebi	340739 310881	-0.0458+-0.0030 **	Admixture from S. cebifrons into S. scrofa S. China
ScSChina ScEurope Scebi	336247 309529	-0.0414+-0.0033 **	Admixture from S. cebifrons into S. scrofa S. China
ScSChina ScSuma1 Scebi	313400 437527	0.1653+-0.0069 **	Admixture from S. cebifrons into S. scorfa Sumatra
ScSChina ScSuma2 Scebi	308561 434049	0.1690+-0.0070 **	Admixture from S. cebifrons into S. scorfa Sumatra
ScSuma2 Sbarba Scebi	283484 1638803	0.7051+-0.0043 **	Supports phylogeny
ScSuma2 Scele Scebi	314388 1847493	0.7092+-0.0040 **	Supports phylogeny
ScSuma2 ScEuroIt Scebi	476677 322826	-0.1924+-0.0070 **	Admixture from S. cebifrons into S. scorfa Sumatra
ScSuma2 ScEurope Scebi	473436 322515	-0.1896+-0.0070 **	Admixture from S. cebifrons into S. scorfa Sumatra
ScSuma2 ScSuma1 Scebi	97396 96679	-0.0037+-0.0100 NS	Non significant
Sverru Sbarba Scebi	401647 649038	0.2355+-0.0067 **	Supports phylogeny
Sverru Scele Scebi	382679 707036	0.2977+-0.0067 **	Supports phylogeny
Sverru ScEuroIt Scebi	1676329 323546	-0.6764+-0.0025 **	Supports phylogeny
Sverru ScEurope Scebi	1675332 324109	-0.6758+-0.0025 **	Supports phylogeny
Sverru ScSChina Scebi	1652076 328611	-0.6682+-0.0028 **	Supports phylogeny
Sverru ScSuma1 Scebi	1517116 317269	-0.6541+-0.0032 **	Supports phylogeny
Sverru ScSuma2 Scebi	1513253 317535	-0.6531+-0.0033 **	Supports phylogeny
Sbarba Scebi Scele	852061 400843	-0.3601+-0.0030 **	Supports phylogeny
Sbarba ScEuroIt Scele	2371394 190408	-0.8513+-0.0021 **	Supports phylogeny
Sbarba ScEurope Scele	2371888 188768	-0.8526+-0.0021 **	Supports phylogeny
Sbarba ScSuma1 Scele	2191337 241604	-0.8014+-0.0039 **	Supports phylogeny
Scebi ScEuroIt Scele	2013399 242023	-0.7854+-0.0028 **	Supports phylogeny
Scebi ScSuma1 Scele	1852602 325514	-0.7011+-0.0058 **	Supports phylogeny
ScEuroIt ScSuma1 Scele	313445 581083	0.2992+-0.0086 **	Admixture from S. celebensis into S. scorfa Sumatra
ScEurope Scebi Scele	239968 2013780	0.7870+-0.0027 **	Supports phylogeny
ScEurope ScEuroIt Scele	110539 111188	0.0029+-0.0048 NS	Non significant
ScEurope ScSuma1 Scele	310952 580279	0.3022+-0.0087 **	Admixture from S. celebensis into S. scorfa Sumatra
ScNChina Sbarba Scele	206883 2337168	0.8374+-0.0024 **	Supports phylogeny
ScNChina Scebi Scele	266772 1981339	0.7627+-0.0033 **	Supports phylogeny
ScNChina ScEuroIt Scele	356752 291971	-0.0999+-0.0031 **	Admixture from S. celebensis into S. scorfa N. China
ScNChina ScEurope Scele	353642 287639	-0.1029+-0.0030 **	Admixture from S. celebensis into S. scorfa N. China
ScNChina ScSChina Scele	248055 256542	0.0168+-0.0027 **	Admixture from S. celebensis into S. scorfa S. China
ScNChina ScSuma1 Scele	319214 520543	0.2397+-0.0090 **	Admixture from S. celebensis into S. scorfa Sumatra
ScNChina ScSuma2 Scele	314264 508455	0.2360+-0.0090 **	Admixture from S. celebensis into S. scorfa Sumatra
ScNChina Sverru Scele	285645 2088918	0.7594+-0.0019 **	Supports phylogeny
ScSChina Sbarba Scele	210041 2331114	0.8347+-0.0025 **	Supports phylogeny
ScSChina Scebi Scele	270547 1975377	0.7591+-0.0033 **	Supports phylogeny
ScSChina ScEuroIt Scele	379166 305788	-0.1071+-0.0031 **	Admixture from S. celebensis into S. scorfa S. China
ScSChina ScEurope Scele	376034 301511	-0.1100+-0.0030 **	Admixture from S. celebensis into S. scorfa S. China
ScSChina ScSuma1 Scele	320899 513395	0.2307+-0.0089 **	Admixture from S. celebensis into S. scorfa Sumatra
ScSChina ScSuma2 Scele	316391 501701	0.2265+-0.0089 **	Admixture from S. celebensis into S. scorfa Sumatra
ScSuma2 Sbarba Scele	238463 2186276	0.8033+-0.0038 **	Supports phylogeny

ScSuma2 Scebi Scele	321090 1847517	0.7039+-0.0058 **	Supports phylogeny
ScSuma2 ScEuroIt Scele	568704 309778	-0.2947+-0.0086 **	Admixture from S. celebensis into S. scorfa Sumatra
ScSuma2 ScEurope Scele	566708 306453	-0.2981+-0.0086 **	Admixture from S. celebensis into S. scorfa Sumatra
ScSuma2 ScSuma1 Scele	109426 111341	0.0087+-0.0127 NS	Non significant
Sverru Sbarba Scele	454236 975124	0.3644+-0.0076 **	Supports phylogeny
Sverru Scebi Scele	650834 707044	0.0414+-0.0089 **	Supports phylogeny
Sverru ScEuroIt Scele	2136868 264353	-0.7798+-0.0017 **	Supports phylogeny
Sverru ScEurope Scele	2138326 262960	-0.7810+-0.0017 **	Supports phylogeny
Sverru ScSChina Scele	2081255 288994	-0.7561+-0.0019 **	Supports phylogeny
Sverru ScSuma1 Scele	1897997 299827	-0.7272+-0.0028 **	Supports phylogeny
Sverru ScSuma2 Scele	1894739 298073	-0.7281+-0.0028 **	Supports phylogeny
Sbarba Scebi ScEuroIt	288268 271408	-0.0301+-0.0032 **	Admixture from S. scrofa Europe into S. barbatus
Sbarba Scele ScEuroIt	283607 190376	-0.1967+-0.0037 **	Admixture from S. scrofa Europe into S. barbatus
Sbarba ScEurope ScEuroIt	105857 2694997	0.9244+-0.0015 **	Supports phylogeny
Sbarba ScSuma1 ScEuroIt	329486 1468100	0.6334+-0.0047 **	Supports phylogeny
Scebi ScSuma1 ScEuroIt	327859 1514415	0.6441+-0.0049 **	Supports phylogeny
Scele Scebi ScEuroIt	242019 320385	0.1393+-0.0032 **	Admixture from S. scrofa Europe into S. cebifrons
Scele ScSuma1 ScEuroIt	313445 1709851	0.6902+-0.0043 **	Supports phylogeny
ScEurope Scebi ScEuroIt	2770099 104799	-0.9271+-0.0014 **	Supports phylogeny
ScEurope Scele ScEuroIt	3095623 111189	-0.9307+-0.0014 **	Supports phylogeny
ScEurope ScSuma1 ScEuroIt	1729451 174284	-0.8169+-0.0026 **	Supports phylogeny
ScNChina Sbarba ScEuroIt	1779651 299588	-0.7118+-0.0044 **	Supports phylogeny
ScNChina Scebi ScEuroIt	1829490 294771	-0.7225+-0.0043 **	Supports phylogeny
ScNChina Scele ScEuroIt	2065345 291965	-0.7523+-0.0039 **	Supports phylogeny
ScNChina ScEurope ScEuroIt	206695 1375324	0.7387+-0.0039 **	Supports phylogeny
ScNChina ScSChina ScEuroIt	491859 401924	-0.1006+-0.0050 **	Admixture from S. scrofa Europe into S. scrofa N. China
ScNChina ScSuma1 ScEuroIt	815916 444794	-0.2944+-0.0041 **	Supports phylogeny
ScNChina ScSuma2 ScEuroIt	799252 441432	-0.2884+-0.0041 **	Supports phylogeny
ScNChina Sverru ScEuroIt	1967828 301289	-0.7344+-0.0044 **	Supports phylogeny
ScSChina Sbarba ScEuroIt	1708646 316175	-0.6877+-0.0042 **	Supports phylogeny
ScSChina Scebi ScEuroIt	1754561 310881	-0.6990+-0.0042 **	Supports phylogeny
ScSChina Scele ScEuroIt	1982051 305779	-0.7327+-0.0038 **	Supports phylogeny
ScSChina ScEurope ScEuroIt	201735 1453858	0.7563+-0.0038 **	Supports phylogeny
ScSChina ScSuma1 ScEuroIt	744943 466742	-0.2296+-0.0039 **	Supports phylogeny
ScSChina ScSuma2 ScEuroIt	729782 463803	-0.2228+-0.0039 **	Supports phylogeny
ScSuma2 Sbarba ScEuroIt	1466463 324879	-0.6373+-0.0047 **	Supports phylogeny
ScSuma2 Scebi ScEuroIt	1511921 322823	-0.6481+-0.0048 **	Supports phylogeny
ScSuma2 Scele ScEuroIt	1708405 309772	-0.6930+-0.0042 **	Supports phylogeny
ScSuma2 ScEurope ScEuroIt	172242 1702124	0.8162+-0.0027 **	Supports phylogeny
ScSuma2 ScSuma1 ScEuroIt	132976 130949	-0.0077+-0.0093 NS	Non significant
Sverru Sbarba ScEuroIt	299702 323488	0.0382+-0.0043 **	Admixture from S. scrofa Europe into S. barbatus
Sverru Scebi ScEuroIt	320135 323542	0.0053+-0.0041 NS	Non significant
Sverru Scele ScEuroIt	343819 264351	-0.1307+-0.0039 **	Admixture from S. scrofa Europe into S. verrucosus
Sverru ScEurope ScEuroIt	110074 2972010	0.9286+-0.0015 **	Supports phylogeny
Sverru ScSChina ScEuroIt	317274 1887843	0.7122+-0.0043 **	Supports phylogeny
Sverru ScSuma1 ScEuroIt	313320 1607551	0.6738+-0.0046 **	Supports phylogeny
Sverru ScSuma2 ScEuroIt	310085 1606873	0.6765+-0.0046 **	Supports phylogeny
Sbarba Scebi ScEurope	287356 271594	-0.0282+-0.0032 **	Admixture from S. scrofa Europe into S. barbatus
Sbarba Scele ScEurope	284167 188745	-0.2018+-0.0036 **	Admixture from S. scrofa Europe into S. barbatus

Sbarba ScEuroIt ScEurope	107496 2695002	0.9233+-0.0015 **	Supports phylogeny
Sbarba ScSuma1 ScEurope	328893 1461394	0.6326+-0.0047 **	Supports phylogeny
Scebi ScEuroIt ScEurope	107568 2770044	0.9252+-0.0015 **	Supports phylogeny
Scebi ScSuma1 ScEurope	328286 1507481	0.6423+-0.0049 **	Supports phylogeny
Scele Scebi ScEurope	239968 321672	0.1455+-0.0033 **	Admixture from S. scrofa Europe into S. cebifrons
Scele ScEuroIt ScEurope	110539 3095569	0.9310+-0.0014 **	Supports phylogeny
Scele ScSuma1 ScEurope	310952 1704828	0.6915+-0.0042 **	Supports phylogeny
ScEuroIt ScSuma1 ScEurope	1729451 173859	-0.8173+-0.0027 **	Supports phylogeny
ScNChina Sbarba ScEurope	1772357 297587	-0.7125+-0.0043 **	Supports phylogeny
ScNChina Scebi ScEurope	1821567 293652	-0.7223+-0.0043 **	Supports phylogeny
ScNChina Scele ScEurope	2059263 287635	-0.7549+-0.0039 **	Supports phylogeny
ScNChina ScEuroIt ScEurope	208644 1375326	0.7366+-0.0037 **	Supports phylogeny
ScNChina ScSChina ScEurope	486682 400863	-0.0967+-0.0049 **	Admixture from S. scrofa Europe into S. scrofa N. China
ScNChina ScSuma1 ScEurope	810946 440281	-0.2962+-0.0039 **	Supports phylogeny
ScNChina ScSuma2 ScEurope	793737 438466	-0.2883+-0.0040 **	Supports phylogeny
ScNChina Sverru ScEurope	1961158 297603	-0.7365+-0.0044 **	Supports phylogeny
ScSChina Sbarba ScEurope	1705950 313930	-0.6892+-0.0042 **	Supports phylogeny
ScSChina Scebi ScEurope	1751600 309535	-0.6996+-0.0042 **	Supports phylogeny
ScSChina Scele ScEurope	1981031 301502	-0.7358+-0.0037 **	Supports phylogeny
ScSChina ScEuroIt ScEurope	207551 1453857	0.7502+-0.0037 **	Supports phylogeny
ScSChina ScSuma1 ScEurope	743510 461045	-0.2345+-0.0037 **	Supports phylogeny
ScSChina ScSuma2 ScEurope	727784 459642	-0.2258+-0.0038 **	Supports phylogeny
ScSuma2 Sbarba ScEurope	1461709 323496	-0.6376+-0.0047 **	Supports phylogeny
ScSuma2 Scebi ScEurope	1507075 322515	-0.6474+-0.0048 **	Supports phylogeny
ScSuma2 Scele ScEurope	1705243 306447	-0.6953+-0.0041 **	Supports phylogeny
ScSuma2 ScEuroIt ScEurope	174006 1702123	0.8145+-0.0027 **	Supports phylogeny
ScSuma2 ScSuma1 ScEurope	133039 129464	-0.0136+-0.0097 NS	Non significant
Sverru Sbarba ScEurope	298164 323382	0.0406+-0.0043 **	Admixture from S. scrofa Europe into S. barbatus
Sverru Scebi ScEurope	318156 324105	0.0093+-0.0040 NS	Non significant
Sverru Scele ScEurope	343447 262953	-0.1327+-0.0040 **	Admixture from S. scrofa Europe into S. verrucosus
Sverru ScEuroIt ScEurope	109969 2972007	0.9286+-0.0015 **	Supports phylogeny
Sverru ScSChina ScEurope	313283 1886033	0.7151+-0.0043 **	Supports phylogeny
Sverru ScSuma1 ScEurope	310980 1601644	0.6748+-0.0046 **	Supports phylogeny
Sverru ScSuma2 ScEurope	307252 1603066	0.6783+-0.0045 **	Supports phylogeny
Sbarba Scebi ScNChina	293298 274455	-0.0332+-0.0037 **	Admixture from S. scrofa N. China into S. barbatus
Sbarba Scele ScNChina	270965 206884	-0.1341+-0.0033 **	Admixture from S. scrofa N. China into S. barbatus
Sbarba ScEuroIt ScNChina	320981 1779648	0.6944+-0.0050 **	Supports phylogeny
Sbarba ScEurope ScNChina	317184 1772356	0.6964+-0.0050 **	Supports phylogeny
Sbarba ScSuma1 ScNChina	311559 1625788	0.6784+-0.0042 **	Supports phylogeny
Scebi ScEuroIt ScNChina	319583 1829474	0.7026+-0.0048 **	Supports phylogeny
Scebi ScSuma1 ScNChina	313220 1673499	0.6847+-0.0043 **	Supports phylogeny
Scele Scebi ScNChina	266772 313218	0.0801+-0.0037 **	Admixture from S. scrofa N. China into S. cebifrons
Scele ScEuroIt ScNChina	356752 2065331	0.7054+-0.0049 **	Supports phylogeny
Scele ScSuma1 ScNChina	319214 1845627	0.7051+-0.0040 **	Supports phylogeny
ScEuroIt ScSuma1 ScNChina	815916 639301	-0.1214+-0.0053 **	Supports phylogeny
ScEurope Scebi ScNChina	1821567 315208	-0.7050+-0.0048 **	Supports phylogeny
ScEurope Scele ScNChina	2059263 353624	-0.7069+-0.0048 **	Supports phylogeny
ScEurope ScEuroIt ScNChina	208644 206697	-0.0047+-0.0087 NS	Non significant
ScEurope ScSuma1 ScNChina	810946 633974	-0.1225+-0.0052 **	Supports phylogeny

ScSChina Sbarba ScNChina	2236585 238273	-0.8074+-0.0026 **	Supports phylogeny
ScSChina Scebi ScNChina	2288585 236160	-0.8129+-0.0025 **	Supports phylogeny
ScSChina Scele ScNChina	2496102 248117	-0.8192+-0.0026 **	Supports phylogeny
ScSChina ScEuroIt ScNChina	1034207 491945	-0.3553+-0.0045 **	Supports phylogeny
ScSChina ScEurope ScNChina	1025627 486772	-0.3563+-0.0045 **	Supports phylogeny
ScSChina ScSuma1 ScNChina	1133275 413736	-0.4651+-0.0033 **	Supports phylogeny
ScSChina ScSuma2 ScNChina	1117189 411875	-0.4613+-0.0031 **	Supports phylogeny
ScSuma2 Sbarba ScNChina	1623368 306439	-0.6824+-0.0041 **	Supports phylogeny
ScSuma2 Scebi ScNChina	1671010 307961	-0.6888+-0.0042 **	Supports phylogeny
ScSuma2 Scele ScNChina	1843437 314258	-0.7087+-0.0039 **	Supports phylogeny
ScSuma2 ScEuroIt ScNChina	636791 799267	0.1131+-0.0051 **	Supports phylogeny
ScSuma2 ScEurope ScNChina	630516 793751	0.1146+-0.0051 **	Supports phylogeny
ScSuma2 ScSuma1 ScNChina	152441 146516	-0.0198+-0.0158 NS	Non significant
Sverru Sbarba ScNChina	308695 322633	0.0221+-0.0045 **	Admixture from S. scrofa N. China into S. barbatus
Sverru Scebi ScNChina	334604 326460	-0.0123+-0.0049 NS	Admixture from S. scrofa N. China into S. verrucosus
Sverru Scele ScNChina	343679 285602	-0.0923+-0.0040 **	Admixture from S. scrofa N. China into S. verrucosus
Sverru ScEuroIt ScNChina	342751 1967805	0.7033+-0.0052 **	Supports phylogeny
Sverru ScEurope ScNChina	339273 1961138	0.7050+-0.0052 **	Supports phylogeny
Sverru ScSChina ScNChina	246770 2410270	0.8143+-0.0028 **	Supports phylogeny
Sverru ScSuma1 ScNChina	303859 1752107	0.7044+-0.0041 **	Supports phylogeny
Sverru ScSuma2 ScNChina	299839 1750728	0.7076+-0.0040 **	Supports phylogeny
Sbarba Scebi ScSChina	294408 275760	-0.0327+-0.0036 **	Admixture from S. scrofa S. China into S. barbatus
Sbarba Scele ScSChina	269768 210046	-0.1245+-0.0033 **	Admixture from S. scrofa S. China into S. barbatus
Sbarba ScEuroIt ScSChina	340024 1708635	0.6681+-0.0050 **	Supports phylogeny
Sbarba ScEurope ScSChina	335976 1705935	0.6709+-0.0050 **	Supports phylogeny
Sbarba ScSuma1 ScSChina	310454 1647960	0.6830+-0.0043 **	Supports phylogeny
Scebi ScEuroIt ScSChina	340739 1754533	0.6748+-0.0048 **	Supports phylogeny
Scebi ScSuma1 ScSChina	313400 1695372	0.6880+-0.0043 **	Supports phylogeny
Scele Scebi ScSChina	270547 313049	0.0728+-0.0038 **	Admixture from S. scrofa S. China into S. cebifrons
Scele ScEuroIt ScSChina	379166 1982030	0.6788+-0.0049 **	Supports phylogeny
Scele ScSuma1 ScSChina	320899 1863914	0.7062+-0.0041 **	Supports phylogeny
ScEuroIt ScSuma1 ScSChina	744943 688939	-0.0391+-0.0049 **	Supports phylogeny
ScEurope Scebi ScSChina	1751600 336247	-0.6779+-0.0048 **	Supports phylogeny
ScEurope Scele ScSChina	1981031 376011	-0.6809+-0.0049 **	Supports phylogeny
ScEurope ScEuroIt ScSChina	207551 201740	-0.0142+-0.0089 NS	Non significant
ScEurope ScSuma1 ScSChina	743510 682655	-0.0427+-0.0048 **	Supports phylogeny
ScNChina Sbarba ScSChina	2236585 240407	-0.8059+-0.0027 **	Supports phylogeny
ScNChina Scebi ScSChina	2288585 240041	-0.8101+-0.0026 **	Supports phylogeny
ScNChina Scele ScSChina	2496102 256614	-0.8136+-0.0027 **	Supports phylogeny
ScNChina ScEuroIt ScSChina	1034207 402014	-0.4402+-0.0036 **	Supports phylogeny
ScNChina ScEurope ScSChina	1025627 400955	-0.4379+-0.0036 **	Supports phylogeny
ScNChina ScSuma1 ScSChina	1133275 442824	-0.4381+-0.0033 **	Supports phylogeny
ScNChina ScSuma2 ScSChina	1117189 439899	-0.4350+-0.0033 **	Supports phylogeny
ScNChina Sverru ScSChina	2410685 251211	-0.8113+-0.0029 **	Supports phylogeny
ScSuma2 Sbarba ScSChina	1645443 305697	-0.6866+-0.0042 **	Supports phylogeny
ScSuma2 Scebi ScSChina	1692673 308586	-0.6916+-0.0042 **	Supports phylogeny
ScSuma2 Scele ScSChina	1861593 316384	-0.7095+-0.0040 **	Supports phylogeny
ScSuma2 ScEuroIt ScSChina	686477 729803	0.0306+-0.0049 **	Supports phylogeny
ScSuma2 ScEurope ScSChina	679291 727799	0.0345+-0.0048 **	Supports phylogeny

ScSuma2 ScSuma1 ScSChina	153703 149279	-0.0146+-0.0149 NS	Non significant
Sverru Sbarba ScSChina	310256 323558	0.0210+-0.0044 **	Admixture from S. scrofa S. China into S. barbatus
Sverru Scebi ScSChina	336656 328609	-0.0121+-0.0049 NS	Non significant
Sverru Scele ScSChina	343332 288989	-0.0859+-0.0040 **	Admixture from S. scrofa S. China into S. verrucosus
Sverru ScEuroIt ScSChina	362923 1887843	0.6775+-0.0052 **	Supports phylogeny
Sverru ScEurope ScSChina	359085 1886035	0.6801+-0.0052 **	Supports phylogeny
Sverru ScSuma1 ScSChina	303204 1772557	0.7079+-0.0041 **	Supports phylogeny
Sverru ScSuma2 ScSChina	299984 1771196	0.7103+-0.0040 **	Supports phylogeny
Sbarba Scebi ScSuma1	334617 284283	-0.0813+-0.0042 **	Admixture from S. scrofa Sumatra into S. barbatus
Sbarba Scele ScSuma1	283262 241556	-0.0795+-0.0042 **	Admixture from S. scrofa Sumatra into S. barbatus
Sbarba ScEuroIt ScSuma1	508064 1468109	0.4858+-0.0108 **	Supports phylogeny
Sbarba ScEurope ScSuma1	505814 1461397	0.4858+-0.0109 **	Supports phylogeny
Scebi ScEuroIt ScSuma1	479254 1514416	0.5192+-0.0095 **	Supports phylogeny
Scele Scebi ScSuma1	325548 315008	-0.0165+-0.0062 NS	Non significant
Scele ScEuroIt ScSuma1	581109 1709853	0.4927+-0.0108 **	Supports phylogeny
ScEurope Scebi ScSuma1	1507491 476813	-0.5194+-0.0095 **	Supports phylogeny
ScEurope Scele ScSuma1	1704832 580275	-0.4921+-0.0108 **	Supports phylogeny
ScEurope ScEuroIt ScSuma1	173844 174273	0.0012+-0.0065 NS	Non significant
ScNChina Sbarba ScSuma1	1625849 468913	-0.5523+-0.0096 **	Supports phylogeny
ScNChina Scebi ScSuma1	1673580 441506	-0.5825+-0.0083 **	Supports phylogeny
ScNChina Scele ScSuma1	1845708 520554	-0.5600+-0.0097 **	Supports phylogeny
ScNChina ScEuroIt ScSuma1	639326 444816	-0.1794+-0.0039 **	Admixture from S. scrofa Sumatra into S. scorfa N. China
ScNChina ScEurope ScSuma1	633998 440304	-0.1803+-0.0039 **	Admixture from S. scrofa Sumatra into S. scorfa N. China
ScNChina ScSChina ScSuma1	413642 442717	0.0340+-0.0031 **	Admixture from S. scrofa Sumatra into S. scorfa S. China
ScNChina ScSuma2 ScSuma1	146514 1965662	0.8613+-0.0032 **	Supports phylogeny
ScNChina Sverru ScSuma1	1752200 567866	-0.5105+-0.0123 **	Supports phylogeny
ScSChina Sbarba ScSuma1	1648027 465660	-0.5594+-0.0095 **	Supports phylogeny
ScSChina Scebi ScSuma1	1695478 437566	-0.5897+-0.0082 **	Supports phylogeny
ScSChina Scele ScSuma1	1864012 513415	-0.5681+-0.0096 **	Supports phylogeny
ScSChina ScEuroIt ScSuma1	688982 466761	-0.1923+-0.0035 **	Admixture from S. scrofa Sumatra into S. scorfa S. China
ScSChina ScEurope ScSuma1	682701 461073	-0.1938+-0.0036 **	Admixture from S. scrofa Sumatra into S. scorfa S. China
ScSChina ScSuma2 ScSuma1	149309 1932600	0.8566+-0.0032 **	Supports phylogeny
ScSuma2 Sbarba ScSuma1	3044648 101747	-0.9353+-0.0019 **	Supports phylogeny
ScSuma2 Scebi ScSuma1	3133950 96793	-0.9401+-0.0017 **	Supports phylogeny
ScSuma2 Scele ScSuma1	3295615 111435	-0.9346+-0.0020 **	Supports phylogeny
ScSuma2 ScEuroIt ScSuma1	2148112 131011	-0.8850+-0.0023 **	Supports phylogeny
ScSuma2 ScEurope ScSuma1	2142807 129526	-0.8860+-0.0023 **	Supports phylogeny
Sverru Sbarba ScSuma1	395324 323272	-0.1003+-0.0095 **	Admixture from S. scrofa Sumatra into S. verrucosus
Sverru Scebi ScSuma1	445444 317266	-0.1681+-0.0108 **	Admixture from S. scrofa Sumatra into S. verrucosus
Sverru Scele ScSuma1	422258 299819	-0.1696+-0.0079 **	Admixture from S. scrofa Sumatra into S. verrucosus
Sverru ScEuroIt ScSuma1	620358 1607555	0.4431+-0.0135 **	Supports phylogeny
Sverru ScEurope ScSuma1	618846 1601649	0.4426+-0.0135 **	Supports phylogeny
Sverru ScSChina ScSuma1	562549 1772558	0.5182+-0.0121 **	Supports phylogeny
Sverru ScSuma2 ScSuma1	120444 3156202	0.9265+-0.0026 **	Supports phylogeny
Sbarba Scebi ScSuma2	332066 283515	-0.0789+-0.0041 **	Admixture from S. scrofa Sumatra into S. barbatus
Sbarba Scele ScSuma2	281842 238472	-0.0834+-0.0042 **	Admixture from S. scrofa Sumatra into S. barbatus
Sbarba ScEuroIt ScSuma2	500835 1466468	0.4908+-0.0107 **	Supports phylogeny
Sbarba ScEurope ScSuma2	497933 1461715	0.4918+-0.0108 **	Supports phylogeny
Sbarba ScSuma1 ScSuma2	100792 3044257	0.9359+-0.0019 **	Supports phylogeny

Scebi ScEuroIt ScSuma2	476677 1511916	0.5206+-0.0094 **	Supports phylogeny
Scebi ScSuma1 ScSuma2	97396 3133520	0.9397+-0.0017 **	Supports phylogeny
Scele Scebi ScSuma2	321090 314401	-0.0105+-0.0060 NS	Non significant
Scele ScEuroIt ScSuma2	568704 1708400	0.5005+-0.0106 **	Supports phylogeny
Scele ScSuma1 ScSuma2	109426 3295155	0.9357+-0.0019 **	Supports phylogeny
ScEuroIt ScSuma1 ScSuma2	132976 2147911	0.8834+-0.0025 **	Supports phylogeny
ScEurope Scebi ScSuma2	1507075 473432	-0.5219+-0.0095 **	Supports phylogeny
ScEurope Scele ScSuma2	1705243 566686	-0.5011+-0.0107 **	Supports phylogeny
ScEurope ScEuroIt ScSuma2	174006 172245	-0.0051+-0.0067 NS	Non significant
ScEurope ScSuma1 ScSuma2	133039 2142602	0.8831+-0.0024 **	Supports phylogeny
ScNChina Sbarba ScSuma2	1623368 461339	-0.5574+-0.0094 **	Supports phylogeny
ScNChina Scebi ScSuma2	1671010 437871	-0.5847+-0.0082 **	Supports phylogeny
ScNChina Scele ScSuma2	1843437 508470	-0.5676+-0.0095 **	Supports phylogeny
ScNChina ScEuroIt ScSuma2	636791 441450	-0.1812+-0.0036 **	Admixture from S. scrofa Sumatra into S.scrofa N. China
ScNChina ScEurope ScSuma2	630516 438486	-0.1796+-0.0036 **	Admixture from S. scrofa Sumatra into S.scrofa N. China
ScNChina ScSChina ScSuma2	411804 439796	0.0329+-0.0031 **	Admixture from S. scrofa Sumatra into S.scrofa S. China
ScNChina ScSuma1 ScSuma2	152441 1965670	0.8561+-0.0035 **	Supports phylogeny
ScNChina Sverru ScSuma2	1750845 552435	-0.5203+-0.0118 **	Supports phylogeny
ScSChina Sbarba ScSuma2	1645443 458243	-0.5643+-0.0093 **	Supports phylogeny
ScSChina Scebi ScSuma2	1692673 434080	-0.5918+-0.0081 **	Supports phylogeny
ScSChina Scele ScSuma2	1861593 501711	-0.5754+-0.0094 **	Supports phylogeny
ScSChina ScEuroIt ScSuma2	686477 463821	-0.1936+-0.0036 **	Admixture from S. scrofa Sumatra into S.scrofa S. China
ScSChina ScEurope ScSuma2	679291 459665	-0.1928+-0.0037 **	Admixture from S. scrofa Sumatra into S.scrofa S. China
ScSChina ScSuma1 ScSuma2	153703 1932602	0.8527+-0.0035 **	Supports phylogeny
Sverru Sbarba ScSuma2	388622 322441	-0.0931+-0.0088 **	Admixture from S. scrofa Sumatra into S. verrucosus
Sverru Scebi ScSuma2	437594 317530	-0.1590+-0.0100 **	Admixture from S. scrofa Sumatra into S. verrucosus
Sverru Scele ScSuma2	416354 298066	-0.1656+-0.0072 **	Admixture from S. scrofa Sumatra into S. verrucosus
Sverru ScEuroIt ScSuma2	604261 1606873	0.4534+-0.0131 **	Supports phylogeny
Sverru ScEurope ScSuma2	601799 1603068	0.4541+-0.0131 **	Supports phylogeny
Sverru ScSChina ScSuma2	547604 1771197	0.5277+-0.0117 **	Supports phylogeny
Sverru ScSuma1 ScSuma2	115659 3156201	0.9293+-0.0023 **	Supports phylogeny
Sbarba Scebi Sverru	560202 401653	-0.1648+-0.0042 **	Admixture from S. verrucosus into S. barbatus
Sbarba Scele Sverru	361747 454241	0.1134+-0.0030 **	Admixture from S. verrucosus into S. celebensis
Sbarba ScEuroIt Sverru	1785476 299700	-0.7125+-0.0026 **	Supports phylogeny
Sbarba ScEurope Sverru	1784516 298168	-0.7137+-0.0025 **	Supports phylogeny
Sbarba ScSuma1 Sverru	1605847 395322	-0.6049+-0.0071 **	Supports phylogeny
Scebi ScEuroIt Sverru	1676329 320136	-0.6793+-0.0033 **	Supports phylogeny
Scebi ScSuma1 Sverru	1517116 445434	-0.5461+-0.0091 **	Supports phylogeny
Scele Scebi Sverru	650834 382681	-0.2595+-0.0040 **	Admixture from S. verrucosus into S. celebensis
Scele ScEuroIt Sverru	2136868 343820	-0.7228+-0.0025 **	Supports phylogeny
Scele ScSuma1 Sverru	1897997 422250	-0.6360+-0.0063 **	Supports phylogeny
ScEuroIt ScSuma1 Sverru	313320 620349	0.3288+-0.0116 **	Admixture from S. verrucosus into S. scrofa Sumatra
ScEurope Scebi Sverru	318156 1675326	0.6808+-0.0033 **	Supports phylogeny
ScEurope Scele Sverru	343447 2138312	0.7232+-0.0025 **	Supports phylogeny
ScEurope ScEuroIt Sverru	109969 110073	0.0005+-0.0046 NS	Non significant
ScEurope ScSuma1 Sverru	310980 618843	0.3311+-0.0116 **	Admixture from S. verrucosus into S. scrofa Sumatra
ScNChina Sbarba Sverru	308695 1763163	0.7020+-0.0030 **	Supports phylogeny
ScNChina Scebi Sverru	334604 1655797	0.6638+-0.0038 **	Supports phylogeny
ScNChina Scele Sverru	343679 2088892	0.7174+-0.0028 **	Supports phylogeny

ScNChina ScEuroIt Sverru	342751 301288	-0.0644+-0.0034 **	Admixture from S. verrucosus into S. scrofa N. China
ScNChina ScEurope Sverru	339273 297603	-0.0654+-0.0035 **	Admixture from S. verrucosus into S. scrofa N. China
ScNChina ScSChina Sverru	246770 251121	0.0087+-0.0028 NS	Non significant
ScNChina ScSuma1 Sverru	303859 567827	0.3028+-0.0123 **	Admixture from S. verrucosus into S. scrofa Sumatra
ScNChina ScSuma2 Sverru	299839 552396	0.2963+-0.0117 **	Admixture from S. verrucosus into S. scrofa Sumatra
ScSChina Sbarba Sverru	310256 1759659	0.7002+-0.0030 **	Supports phylogeny
ScSChina Scebi Sverru	336656 1652069	0.6614+-0.0039 **	Supports phylogeny
ScSChina Scele Sverru	343332 2081236	0.7168+-0.0028 **	Supports phylogeny
ScSChina ScEuroIt Sverru	362923 317274	-0.0671+-0.0031 **	Admixture from S. verrucosus into S. scrofa S. China
ScSChina ScEurope Sverru	359085 313285	-0.0681+-0.0032 **	Admixture from S. verrucosus into S. scrofa S. China
ScSChina ScSuma1 Sverru	303204 562540	0.2996+-0.0120 **	Admixture from S. verrucosus into S. scrofa Sumatra
ScSChina ScSuma2 Sverru	299984 547595	0.2921+-0.0115 **	Admixture from S. verrucosus into S. scrofa Sumatra
ScSuma2 Sbarba Sverru	388622 1602480	0.6096+-0.0068 **	Supports phylogeny
ScSuma2 Scebi Sverru	437594 1513247	0.5514+-0.0088 **	Supports phylogeny
ScSuma2 Scele Sverru	416354 1894724	0.6397+-0.0061 **	Supports phylogeny
ScSuma2 ScEuroIt Sverru	604261 310084	-0.3217+-0.0111 **	Admixture from S. verrucosus into S. scrofa Sumatra
ScSuma2 ScEurope Sverru	601799 307254	-0.3240+-0.0111 **	Admixture from S. verrucosus into S. scrofa Sumatra
ScSuma2 ScSuma1 Sverru	115659 120438	0.0202+-0.0265 NS	Non significant

Table S8. Results of D-statistics analysis. P1, P2 represents putative sister taxa, while P3 represents putative admixting taxa. ABBA and BABA count refers to number of match between P1/P3 and P2/P3 respectively. Significance level: * p<0.05; ** p<0.001; NS p > 0.05.
