# Supplementary material for: Genome sequencing reveals fine scale diversification and reticulation history during speciation in Sus
Source: Genome Biol. 2013 Sep 26;14(9):R107. doi: 10.1186/gb-2013-14-9-r107 (PMC4053821; doi:10.1186/gb-2013-14-9-r107)
Supplement: Additional file 6 — Text that contains information about fossil calibration. [file gb-2013-14-9-r107-S6.PDF]

Additional file 6 – Fossil calibration

(references at the end of the text)

Root calibration

The first appearance of the subfamily *Suinae* in Africa is marked by *Kolpochoerus*, discovered at Kossom Bougoudi, Chad[1] near the Plio-Miocene boundary 5-5.5 Mya. However, the monophyletic status of African Suinae is controversial. It has been suggested that *Kolpocheorus* and *Metridiocoerus* gave rise to *Hylochoerus* and *Phacochoerus* respectively and that *Potamochoerus* evolved as a separate lineage[2]. The first appearance of *Metridiochoerus* and *Phacochoerus* in the fossil record lies in the Shungura formation of Ethopia[3] (2.6-1.8 Mya). However, this time is likely to be too low to be used in our study. Moreover, the monophylic relationship of extant African *Suinae* have been suggested in a recent phylogenetic study[4] that place the age of the nodes *Sus* vs African *Suinae* at 7-14Mya. Therefore, we decided to use 5.5 Mya as a minimum soft bound for the calibration of the root of our tree.

S. verrucosus' node

For evolution of Suids on Java, Hardjasasmita[5] considered two possibilities: (1) *S. brachygnathus* is ancestral to *S. macrognathus*; (2) *S. macrognathus* evolved somewhere outside Java and immigrated together with several new faunal elements[7]. Hardjasasmita favoured the former possibility, and he hypothesized that *S. brachygnathus* became extinct after the arrival of *S. scrofa*[6]. Based on the shape of the mandible and dental morphology, Aimi (1989) suggested that *S. barbatus* is clearly different from all other taxa (*verrucosus*, *brachygnathus*, *macrognathus*, *terhaari* and *stremmi*). If this is correct, then all Javan species are chronospecies starting with *S. stremmi* in the Late Pliocene / Early Pleistocene, followed by *brachygnathus*, *macrognathus*, and *terhaari*. At the same time, *S. barbatus* would have evolved in the dipterocarp forests of Borneo and possibly Malaysia and Sumatra. Overall our Phylogenomic analysis supports the hypothesis of Text S1Hardjasasmita, as we found that *S. verrucosus* is very distinct to *S. barbatus*. The isolation of *S. verrucosus* on Java seems to be the first divergence to occur in ISEA. Together these evidences support the monophyly of Sus species in Java. The oldest fossil to occur on Java is *S. stremmi* from the Kali Glagah formation ~2Mya. Recent palaeogeographical

reconstruction of Java[7] shows that Java would have been the first island to be reachable, from Borneo and Sumatra, by large mammals ~2MY ago. Taking into account this information, we decided to use a minimum soft bound at 1.5My to calibrate the node of *S. verrucosus*.

Asian / European node

For the last calibration we used the first fossil appearance of *S. scrofa* in Europe, in the late early Pleistocene[8]. We calibrated the divergence between European and Asian *S. scrofa* with a minimum soft bound at 0.8Mya.

References

[1] Brunet, M. & White, T.D. Deux nouvelles espèces de Suini (Mammalia , Suidae) du continent Africain (Éthiopie ; Tchad). *Comptes Rendu de l'Academie des Sciences* 332, 51-57 (2001).

[2] Geraads, D. A. New skulls of Kolpochoerus phacochoeroides (Suidae : Mammalia) from the late Pliocene of Ahl al Oughlam, Morroco. *Palaeontologica Africa* 40, 69-83 (2004).

[3] de Heinzelin, J. The Omo Group: Archives of the International Omo Research Expedition. *Musee Royal de l'Afrique Centrale, Annales Series 8, Tervuren, Belgique* (1983).

[4] Gongora, J. *et al.* Rethinking the evolution of extant sub-Saharan African suids (Suidae, Artiodactyla). *Zoologica Scripta* 40, 327-335 (2011).

[5] Hardjasasmita, H.S. Taxonomy and phylogeny of the Suidae (Mammalia) in Indonesia. *Scripta Geologica* 85, 1-68 (1987).

[6] Aimi, M. A mandible of Sus stremmi Koenigswald, 1933, from Cisaat, Centra Java, Indonesia. *Publications of the Geological Research and Development Center* 4-10 (1989).

[7] Meijaard, E. Solving mammalian riddles. A reconstruction of the Tertiary and Quaternary distribution of mammals and their palaeoenvironments in island South-East Asia. PhD Thesis, The Australian National University, Camberra (2009).

[8] Vandermade, J. Ungulates from Atapuerca TD6. *Journal of Human Evolution* 37, 389-413 (1999).
