# Supplementary material for: Genome sequencing reveals fine scale diversification and reticulation history during speciation in Sus
Source: Genome Biol. 2013 Sep 26;14(9):R107. doi: 10.1186/gb-2013-14-9-r107 (PMC4053821; doi:10.1186/gb-2013-14-9-r107)
Supplement: Additional file 7 — Figure S3 describing the demographic history of the population from MSEA. [file gb-2013-14-9-r107-S7.PDF]

Additional File 7 – PSMC results for Eurasian *S. scrofa*

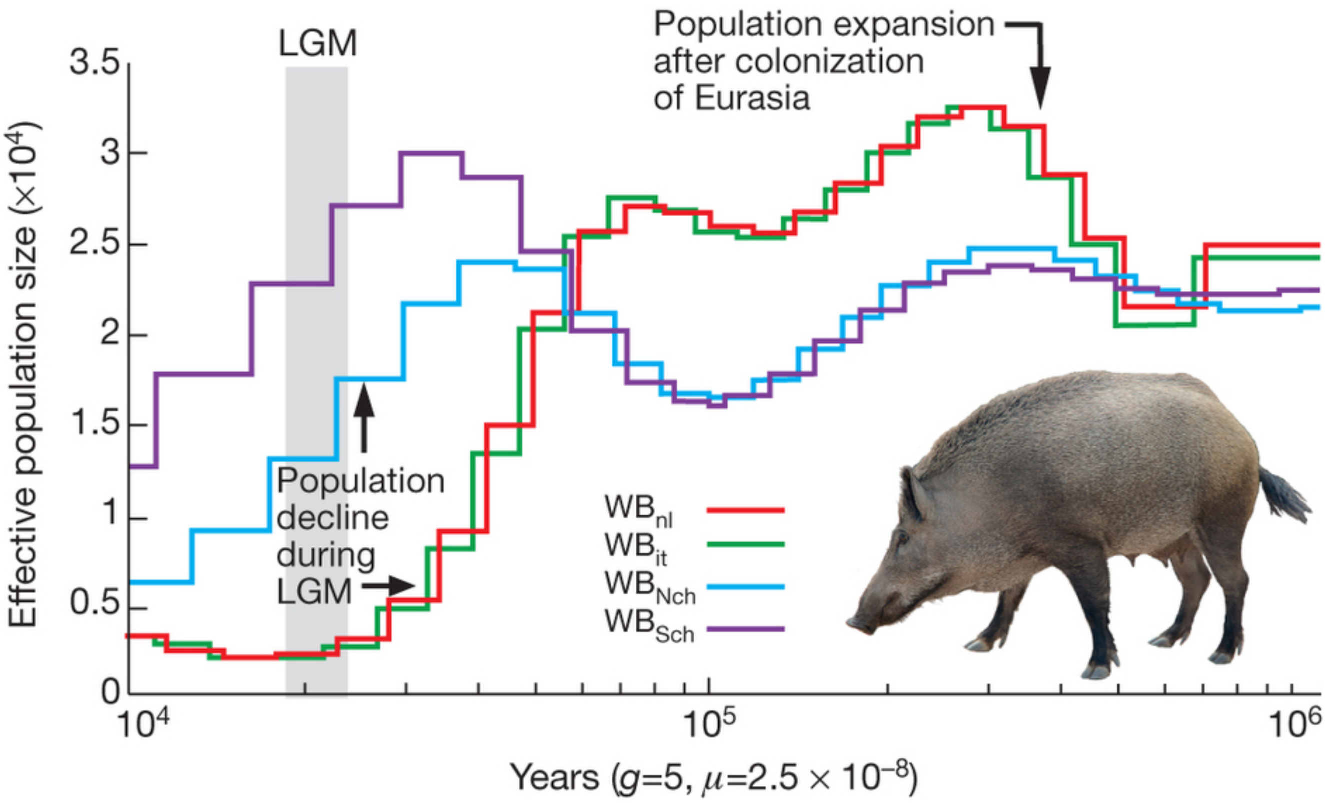

Figure S3: Population size of wild boars in Eurasia, adapted from Groenen *et. al.* 2013 [15]. WBnl = *S. scrofa* Europe (ScEurope); WBit = *S. scrofa* Italy (ScEuroIt); WBNch = *S. scrofa* North China (ScNChina); WBSch = *S. scrofa* South China (ScSChina).
