# Supplementary material for: Genome sequencing reveals fine scale diversification and reticulation history during speciation in Sus
Source: Genome Biol. 2013 Sep 26;14(9):R107. doi: 10.1186/gb-2013-14-9-r107 (PMC4053821; doi:10.1186/gb-2013-14-9-r107)
Supplement: Additional file 8 — Figure S4, a phylogenetic tree constructed using SNPs sequenced with the Illumina Porcine SNP60 array. [file gb-2013-14-9-r107-S8.PDF]

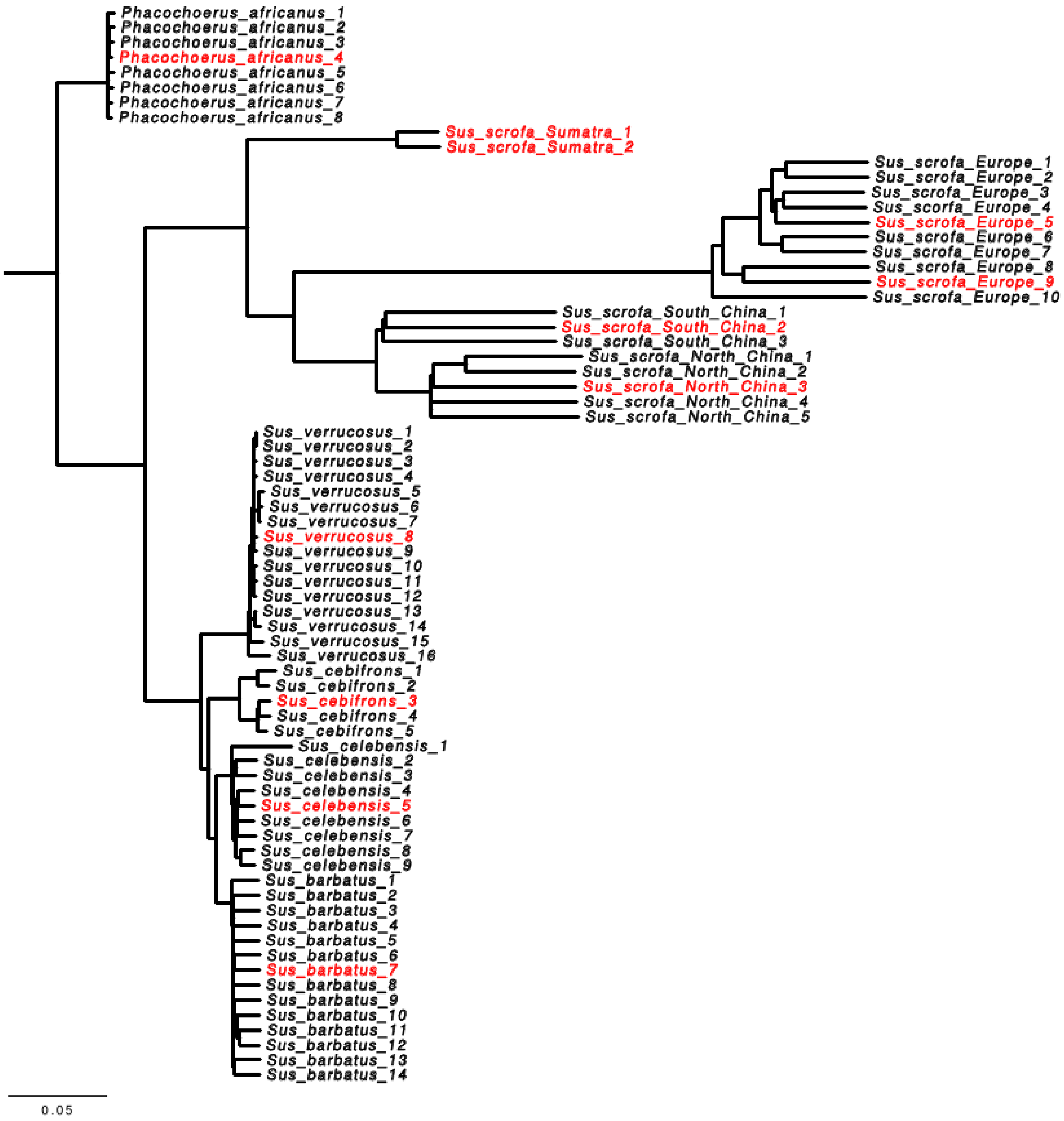

Figure S4: Neighbor-Joining tree based on Ilumina Porcine 60K Bead Chip (Ramos *et. al.* 2009). Individuals highlighted in red correspond to whole-genome samples used in this study. This tree demonstrate that each sample used in this study is clustering with other relatives from the same population / species, and should therefore be representative of their respective population / species.
